# An Efficient and Reliable Statistical Method for Estimating Functional Connectivity in Large Scale Brain Networks Using Partial Correlation

**DOI:** 10.3389/fnins.2016.00123

**Published:** 2016-03-31

**Authors:** Yikai Wang, Jian Kang, Phebe B. Kemmer, Ying Guo

**Affiliations:** ^1^Department of Biostatistics and Bioinformatics, The Rollins School of Public Health, Emory UniversityAtlanta, GA, USA; ^2^Department of Biostatistics, School of Public Health, University of MichiganAnn Arbor, MI, USA

**Keywords:** network analysis, functional connectivity, fMRI, partial correlation, precision matrix, CLIME, L1 regularization

## Abstract

Currently, network-oriented analysis of fMRI data has become an important tool for understanding brain organization and brain networks. Among the range of network modeling methods, partial correlation has shown great promises in accurately detecting true brain network connections. However, the application of partial correlation in investigating brain connectivity, especially in large-scale brain networks, has been limited so far due to the technical challenges in its estimation. In this paper, we propose an efficient and reliable statistical method for estimating partial correlation in large-scale brain network modeling. Our method derives partial correlation based on the precision matrix estimated via Constrained L1-minimization Approach (CLIME), which is a recently developed statistical method that is more efficient and demonstrates better performance than the existing methods. To help select an appropriate tuning parameter for sparsity control in the network estimation, we propose a new *Dens*-based selection method that provides a more informative and flexible tool to allow the users to select the tuning parameter based on the desired sparsity level. Another appealing feature of the *Dens*-based method is that it is much faster than the existing methods, which provides an important advantage in neuroimaging applications. Simulation studies show that the *Dens*-based method demonstrates comparable or better performance with respect to the existing methods in network estimation. We applied the proposed partial correlation method to investigate resting state functional connectivity using rs-fMRI data from the Philadelphia Neurodevelopmental Cohort (PNC) study. Our results show that partial correlation analysis removed considerable between-module marginal connections identified by full correlation analysis, suggesting these connections were likely caused by global effects or common connection to other nodes. Based on partial correlation, we find that the most significant direct connections are between homologous brain locations in the left and right hemisphere. When comparing partial correlation derived under different sparse tuning parameters, an important finding is that the sparse regularization has more shrinkage effects on negative functional connections than on positive connections, which supports previous findings that many of the negative brain connections are due to non-neurophysiological effects. An R package “DensParcorr” can be downloaded from CRAN for implementing the proposed statistical methods.

## Introduction

In recent years, network-oriented analyses have shown great promise for understanding brain organization and its involvement in mental disorders. With the advancement of neuroimaging technologies, the study of whole-brain functional connectivity analysis using functional magnetic resonance imaging (fMRI) data has stimulated an enormous amount of interest (Biswal et al., 1995; Bullmore and Sporns, 2009; Deco et al., 2011; Satterthwaite et al., 2015; Zhang et al., 2015). In particular, there has been a strong focus on investigating intrinsic brain connectivity using resting-state fMRI (rs-fMRI), which measures the spontaneous low-frequency fluctuations in the blood oxygen level dependent (BOLD) signal in subjects at rest (Ogawa et al., 1990; Dosenbach et al., 2010).

Various methods have been proposed for assessing the brain connectivity between selected network nodes. One of the simplest and most frequently used methods in the neuroimaging community is via pairwise correlations between BOLD time courses from two brain network nodes. These correlations are of great interest to neuroscientists in that they can reflect the functional connectivity between brain regions and help explore the overall network structure of the whole brain (Church et al., 2009; Seeley et al., 2009).

However, there are well-known limitations in the correlation analysis. Pearson correlation, which we will henceforth refer to as “full correlation,” only reflects the marginal association between network nodes and is not an appropriate tool for capturing the true or direct functional connection between them. For example, a large correlation between a pair of nodes can appear due to their common connections to a third-party node, even if the two nodes are not directly connected (Smith et al., 2011). Using full correlation, investigators often identify significant connections between a large number of node pairs in brain networks. It is difficult to distinguish which of these significant correlations reflect true functional connections and which are caused by confounding factors such as global effects or third-party nodes.

A network modeling method that has shown great potential in addressing this major issue is partial correlation (Smith, 2012). Partial correlation measures the direct connectivity between two nodes by estimating their correlation after regressing out effects from all the other nodes in the network, hence avoiding spurious effects in network modeling. A partial correlation value of zero implies an absence of direct connections between two nodes given all the other nodes. Through a set of extensive and realistic simulation studies, Smith et al. (2011) compared the performance of a wide range of network modeling methods for fMRI data and found that partial correlation is among the top methods that performed excellently under various types of scenarios and showed high sensitivity to detect true functional connections.

Although it has been shown to have major advantages in studying brain connectivity, the application of partial correlation in the neuroimaging community has been limited. This is mainly because the estimation of partial correlation is more difficult than full correlation. Direct estimation based on the regression approach is inefficient in terms of computational time and often fails due to the multicollinearity among node time series. A more efficient way to estimate the full set of partial correlations is via the inverse of the covariance matrix, also known as the precision matrix (Marrelec et al., 2006), where the off-diagonals of a precision matrix have a one-to-one correspondence with partial correlations (Peng et al., 2009).

Estimation of the precision matrix is not a trivial task since it involves the inversion of the covariance matrix, especially for a large dimensional case. Furthermore, a precision matrix needs to satisfy the positive definite condition which further increases difficulty in its estimation. In neuroimaging applications, this task could become even more challenging because there are often a large number of nodes in brain networks and a limited number of observations at each node (e.g., shorter fMRI scans) (Zhang et al., 2015). Under this setting, estimation of the precision matrix requires a huge computational load and may not be stable. A few methods have been developed for this purpose in the neuroimaging community (Schmittmann et al., 2015). Schäfer and Strimmer (2005) developed a shrinkage approach to estimate the covariance matrix. Moore-Penrose inverse of the covariance matrix can also be applied to directly estimate the precision matrix (Ben-Israel and Greville, 2003). Moreover, the most popular approach is to apply the sparse regularization via the L1 penalty (Meinshausen and Bühlmann, 2006; Friedman et al., 2008; Liu and Luo, 2012) in estimating the precision matrix under the normality assumption (Smith et al., 2011). As an extension, several works were proposed to relax the normality assumption for graphical models (Liu et al., 2012; Han et al., 2013). The existing approaches can become quite time consuming when the dimension of the precision matrix becomes high. Furthermore, based on our experiments, when estimating large-scale brain networks, the existing computational tools used in the community often either have computational issues or lack of accuracy in capturing some key features in brain organization. Finally, the sparse regularization estimation usually requires the selection of a tuning parameter to control the sparsity of the estimated precision matrix, and the results vary significantly depending on the choice. Currently, the selection of the tuning parameter is often fairly subjective in applications.

In this paper, we present a more efficient and reliable statistical procedure for estimating partial correlation in brain network modeling under the regularized precision matrix framework. The proposed procedure first estimates the precision matrix via the Constrained L1-minimization Approach (CLIME) (Cai et al., 2011). Compared with other regularization methods such as Lasso, CLIME is shown to have better theoretical properties as well as computational advantages. Theoretically, CLIME precision matrix estimators are shown to converge to the true precision matrix at a faster rate as compared to the traditional L1 regularization methods. Computationally, CLIME can be easily implemented by linear programming and is scalable to a high dimensional precision matrix with a large number of nodes. As with the other regularization methods, CLIME requires the setting of a tuning parameter for controlling the sparsity. The existing selection methods often face challenges in estimating large-scale brain networks in that they either tend to select overly dense networks or are computationally expensive. To address this issue, we propose a method to provide a systematic approach that allows the users to make a more informed choice of the tuning parameter. Specifically, we propose a *Dens* criterion function that reflects how dense the estimated precision matrix is under various tuning parameters. Then by setting a desired density level one would like to achieve, the users can find the appropriate tuning parameter to use for CLIME. The proposed *Dens*-based selection method is easy to implement, computationally much faster than existing methods, and provides users the flexibility to control the sparsity of the estimated precision matrix. Simulation studies show that our *Dens*-based method demonstrates similar or better accuracy in estimating the precision matrix as compared to the more complicated and computationally expensive selection methods. We also show via a real fMRI data example that the selection of the tuning parameter based on the proposed method is highly consistent across subjects. After estimating the precision matrix using CLIME with the chosen tuning parameter, we provide the formula for deriving the partial correlation matrix from the precision matrix.

We apply the proposed partial correlation estimation procedure to investigate direct brain functional connectivity using resting state fMRI data collected in the Philadelphia Neurodevelopmental Cohort (PNC) study (Satterthwaite et al., 2014). We compare the direct brain connectivity pattern based on partial correlation with the marginal brain connectivity based on full correlation. We examine edges in the brain network that are consistently identified by both the partial correlation and full correlation method vs. edges for which the two methods show inconsistent results. Additionally, we examine how the partial-correlation-based direct connectivity networks change when we impose different levels of sparsity in the estimated network.

## Methods

### Partial correlation: definition and derivation

In this section, we first introduce the concept and definition of partial correlation under the brain network modeling framework. To set notation, let **X** = {X_1_, …, X_M_} denote the fMRI BOLD signal at M nodes (Mx1 vector) in the network in an fMRI scan. Let **X**_t_, t = 1, …, T, denote the T realizations of **X** in fMRI scans obtained during a scanning session. Partial correlation between nodes i and j is defined as the correlation between X_i_ and X_j_ conditioning on all the other nodes, i.e.:

$$\begin{array}{l} \rho_{\text{ij}}=\text{corr}\left({\text{X}}_{\text{i}},{\text{X}}_{\text{j}}{\text{|X}}_{-(\text{i},\text{j})}\right),{\text{X}}_{-(\text{i},\text{j})}=\left\{{\text{X}}_{\text{k}}:\text{1}\leq \text{k}\neq \text{i},\text{j}\leq \text{M}\right\},\\ \text{ i},\text{j}=\text{1},\ldots ,\text{ M},\text{ i}\neq \text{j}. \end{array}$$

In the context of brain networks, partial correlation is the correlation between time series of two nodes, after adjusting for the time series from all other network nodes (Smith et al., 2011). As an example, consider a simple three-node network (M = 3). To derive the partial correlation between nodes 1 and 2, we first regress the time series of node 1 against the time series of node 3 and denote the residual as **R**_1|3_, then regress the time series of node 2 against the time series of node 3 and denote the residual as **R**_2|3_; the partial correlation between node 1 and 2 can then be obtained as the correlation between **R**_1|3_ and **R**_2|3_.

In addition to the derivation based on linear regression, partial correlation can also be derived from the inverse covariance matrix, also known as the precision matrix. Let **Σ** be the MxM covariance matrix based on **X** and let **Ω** = **Σ**^−1^ = {ω_ij_}_MxM_ be the precision matrix. The partial correlation between node i and j can be derived from the precision matrix as Peng et al. (2009):

(1) $$\begin{array}{l} \rho_{\text{ij}}=-\omega_{\text{ij}}∕\sqrt{\omega_{\text{ii}}\omega_{\text{jj}}}. \end{array}$$

Under the Gaussian assumption, one can infer that node i and j are conditionally independent given the other nodes when ρ_ij_ equals 0. Therefore, partial correlation provides a way to assess the direct connection between nodes and allows correct estimation of the true network by removing all the confounding effects (Smith et al., 2011).

To illustrate the difference between full correlation and partial correlation, we provide a toy example using a 3-node network. X_1_, X_2_, X_3_ represent the measurements from the 3 nodes, where

(2) $$\begin{array}{r} {\text{X}}_{1}=\alpha_{1}{\text{X}}_{2}+\varepsilon_{1},{\text{X}}_{3}=\alpha_{2}{\text{X}}_{2}+\varepsilon_{2},\varepsilon_{1},\varepsilon_{2},{\text{X}}_{2}\sim_{\text{iid}}\text{N}\left(0,1\right). \end{array}$$

**Figure 1:**
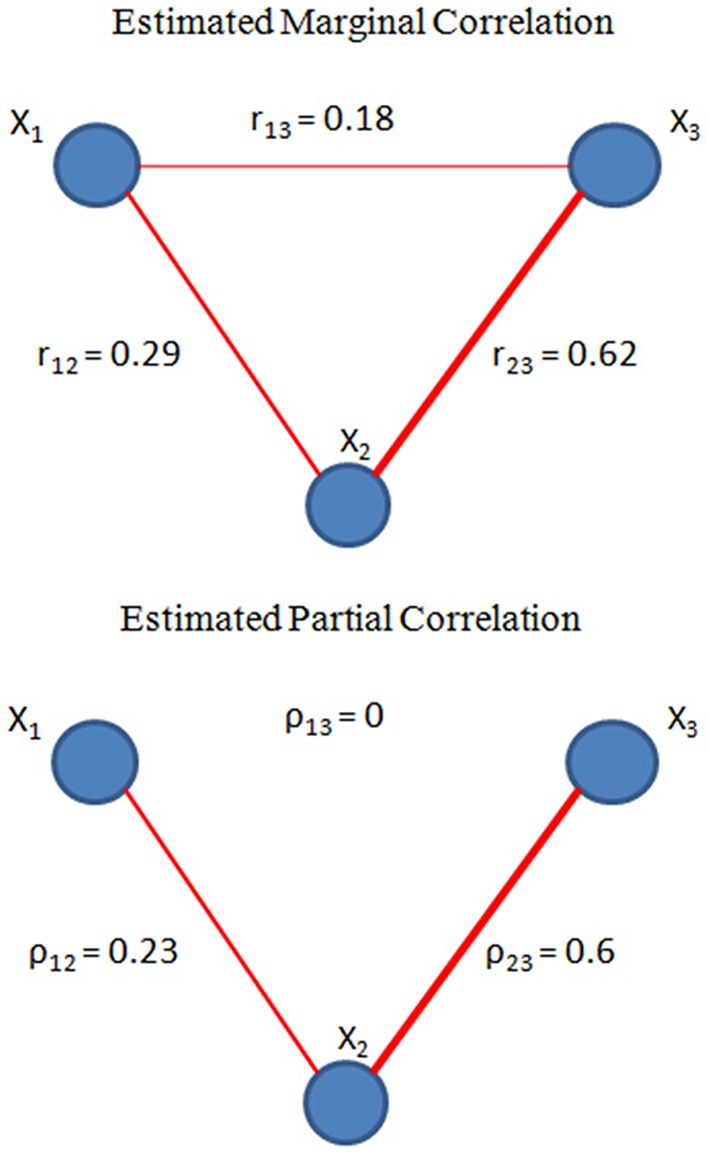
**Toy example on partial correlation (bottom) and full correlation (top)**. Within a 3-node network, where X_1_ = 0.3X_2_ +ε_1_, X_3_ = 0.8X_2_ +ε_2_ and ε_1_, ε_2_, X_2_ ~ _iid_N(0,1), we estimated the connectivity based on full correlation and partial correlation. As shown in the figure, partial correlation can detect the conditional independence between X_1_and X_3_, whereas full correlation only measures the marginal correlation which is resulted from X_2_.

Here both X_1_ and X_3_ are directly associated with X_2_, but X_1_ and X_3_ are not directed related to each other given X_2_. We then estimated both the full correlation and partial correlation based on the time series generated from (2) with α_1_ = 0.3 and α_2_ = 0.8. The results are presented in Figure 1. Both correlation methods were able to detect the true connectivity between nodes 1 and 2, and between nodes 2 and 3. However, for nodes 1 and 3, the full correlation estimate implies that they were also associated. From the data-generating model (2), we know that this association is not due to the true connection between nodes 1 and 3 but rather caused by their common connection with node 2. The partial correlation estimate for this connection had a value of zero, correctly reflecting that there was no direction connection between nodes 1 and 3. This toy example demonstrates the ability of partial correlation in removing spurious associations due to a third-party node, and hence provides a more reliable measure for direct connectivity in brain networks.

### The proposed procedure for estimating partial correlation using neuroimaging data

Unlike full correlation which can be readily calculated from the observed fMRI data, the estimation of partial correlation is less straightforward and more computationally challenging. The precision matrix method provides an efficient way to obtain the full set of partial correlations between all node pairs in a network. However, since estimating the precision matrix commonly involves inverting the covariance matrix, this approach becomes challenging as the number of nodes (and the dimension of the covariance matrix) increases. In particular, direct inversion of the covariance matrix is not feasible when the number of nodes is larger than the number of observations at each node, such as the case of estimating large-scale brain networks in relatively short fMRI scanning sessions. Various approaches based on regularization methods such as Graphical lasso have been applied to address this issue in neuroimaging studies (Friedman et al., 2008). The issues with the existing approaches are that they require long computation time and often fail when the number of nodes is large. Another difficulty is that the regularization methods require the selection of a tuning parameter to control the sparsity of the estimated precision matrix, and in current neuroimaging applications, this selection is often conducted in a fairly subjective manner.

In this section, we propose a new statistical procedure for estimating the partial correlations in a brain network. Our proposed procedure consists of three parts: (1) estimating the precision matrix using Constrained L1-minimization for Inverse Matrix Estimation (CLIME), which is a recently developed statistical method that is computationally more efficient and demonstrates better performance as compared to many existing algorithms; (2) choosing the tuning parameter for the CLIME algorithm based on our proposed *Dens*-based method, which is fast and can be easily understood and controlled by the users; and (3) deriving the full set of partial correlations from the estimated precision matrix.

#### A constrained L1 approach (CLIME) to sparse precision matrix estimation

The CLIME method is an approach that has been recently developed in the statistical community for estimating a sparse precision matrix (Cai et al., 2011). The CLIME estimator of the precision matrix **Ω** is derived using the following procedure. First, we find the solution **Ω**^1^ of the following optimization problems:

(3) $$\Omega^{1}=\text{arg min }||\Omega |{|}_{1}\text{ subject to }|\hat{\Sigma }\Omega -I{|}_{\infty }\leq \lambda ,$$

here, **Ω**^1^ = $\Omega^{1}=\{{\tilde{\omega }}_{\text{ij}}\}\text{MxM}$ is an initial estimator of the precision matrix **Ω**, $\hat{\Sigma }$ is the estimated covariance matrix, λ is a tuning parameter ranging from 0 to 1, where a larger λ imposes a stronger sparsity regularization and hence yields a more sparse **Ω**^1^. Because **Ω**^1^ is not necessarily symmetric, the final CLIME estimator ${\hat{\Omega }}_{*}$ is obtained by symmetrizing **Ω**^1^ as follows.

(4) $$\begin{array}{l} \;{\hat{\Omega }}_{*}={\left\{{\hat{\omega }}_{\text{ij}}\right\}}_{\text{MxM}},\\ \text{with }{\hat{\omega }}_{\text{ij}}=\text{min}({\tilde{\omega }}_{\text{ij}},\;{\tilde{\omega }}_{\text{ji}}). \end{array}$$

A unique feature of the CLIME method is that it develops an approach to solve the convex program (3) by decomposing it into M vector minimization problems that estimate each column of **Ω**^1^ one at a time. It can be shown that solving the optimization problem in (3) is equivalent to solving the M vector minimization problem, which can be achieved via linear programming. By estimating the precision matrix column-by-column, CLIME significantly reduces the computational and statistical difficulties in its estimation. Another appealing feature is that the final CLIME estimator ${\hat{\Omega }}_{*}$ is shown to be positive definite with high probability (Cai et al., 2011). This means that the CLIME method has a high chance of producing a valid precision matrix estimate for brain network modeling.

#### Regularization selection

As with other regularization methods, the CLIME approach also requires the specification of a tuning parameter, i.e., λ in (3). This parameter controls the sparsity of the estimated precision matrix and the subsequent estimate of the partial correlation matrix. An advantage of the CLIME method is that the tuning parameter is selected within the finite range of 0–1, whereas the tuning parameter in other regularization methods does not have a finite range. For example, graphical lasso involves a tuning parameter that ranges from 0 to 8. From (3), a smaller λ yields a denser graph and larger λ yields a sparser graph. When λ approaches toward 1, which means imposing strongest sparsity regularization, Ω^*^ will approach an empty matrix which corresponds to an empty network without any edges. When λ approaches toward 0, the minimum sparsity regularization, ${\hat{\Omega }}_{*}$ will approach the precision matrix estimate that is obtained without the sparsity constraint.

Two common ways to select the tuning parameter in regularization methods are AIC and BIC (Schwarz, 1978; Akaike, 1998). Let ${\hat{\Omega }}_{*}(\lambda )$ be the estimated precision matrix based on tuning parameter λ. AIC selects λ such that:

$$\begin{array}{l} \hat{\lambda }={\text{argmin}}_{\lambda }\left\{-2\text{log}|{\hat{\Omega }}_{*}\left(\lambda \right)|+2\text{trace}\left(\hat{\Sigma }{\hat{\Omega }}_{*}\left(\lambda \right)\right)+2\text{d}\left(\lambda \right)\right\}, \end{array}$$

and BIC selects λ such that:

$$\begin{array}{l} \hat{\lambda }={\text{argmin}}_{\lambda }\left\{-2\text{log}|{\hat{\Omega }}_{*}\left(\lambda \right)|+2\text{trace}\left(\hat{\Sigma }{\hat{\Omega }}_{*}\left(\lambda \right)\right)+\text{d}\left(\lambda \right)\text{log}\left(\text{T}\right)\right\}. \end{array}$$

Here, d(λ) denotes the degrees of freedom of the underlying Gaussian model. The d(λ) is difficult to estimate in the high-dimensional setting where the number of nodes in the network (M) exceeds the number of observations (T) at each node. In this case, the d(λ) is often estimated by the number of non-zero elements in ${\hat{\Omega }}_{*}\left(\lambda \right)$. It has been shown that AIC and BIC methods tend to yield an overly dense precision matrix in the high-dimensional case (Liu et al., 2010).

Another commonly used method for selecting λ is the k-fold cross-validation (K-CV) method (Efron, 1982). In this type of procedure, the observed data are partitioned into k blocks, where k-1 blocks are used as training data to estimate the precision matrix and the remaining block is retained as validation data. For each λ value in the search grid, one estimates the precision matrix and corresponding partial correlations using the k-1 blocks of training data and then evaluates a loss function of the estimates using the validation data. Two typical loss functions are the negative log-likelihood and Trace L2 defined below.

$$\begin{array}{l} \text{Negative log}-\text{likelihood}:-\text{log|}{\hat{\Omega }}_{*}(\lambda )|+\text{trace}\left(\hat{\Sigma }{\hat{\Omega }}_{*}\left(\lambda \right)\right)-\text{M}\\ \text{Trace L2}:\text{ trace}\left(\text{diag}{\left(\hat{\Sigma }{\hat{\Omega }}_{*}\left(\lambda \right)-I_{\text{M}}\right)}^{2}\right) \end{array}$$

The K-CV methods based on these two loss functions are implemented in the CLIME R package (Cai et al., 2012). One issue with K-CV methods is that they are typically computationally expensive. Furthermore, it has been shown that K-CV based on the negative log-likelihood loss function tends to select overly dense graphs (Wasserman and Roeder, 2009).

In this paper, we present a new method for selecting λ. Specifically, we propose a *Dens* criterion function that measures how dense the estimated precision matrix is. Then we consider a series of λ within the finite range (0, 1). We start with a large value of λ which results in an extremely sparse graph with little or no edges, then decrease λ so that the estimated precision matrix becomes denser and more edges are allowed to appear in the graph. We continue to decrease λ until the density of the precision matrix, measured by the proposed criterion function, reaches its plateau and remains stable. Finally, we examine the profile of the *Dens* criterion function across the series of λ values and select the value of λ that corresponds to the desired density level that the investigator would like to achieve.

To measure how dense an estimated precision matrix is, we propose the following *Dens* criterion function:

(5) $$Dens\left(\Omega \right)=\sum_{\text{ij}}|\omega_{\text{ij}}|,\text{where }\Omega =\{\omega_{\text{ij}}\}.$$

That is, *Dens* is the sum of the absolute values of all elements in the estimated precision matrix, and measures the density level of the precision matrix. Essentially, *Dens* is the matrix-wise L1 norm of **Ω**.

For the CLIME procedure, we consider a monotonically decreasing sequence {λ_*n*_, *n* = 0, 1, …} within the range (0,1) with λ_0_ → 1 and λ_*n*_ → 0 as *n* increases. For simplicity, we denote $Dens\left({\hat{\Omega }}_{*}\left(\lambda \right)\right)$ as *Dens*(λ). For λ_0_ → 1, the CLIME estimator ${\hat{\Omega }}_{*}\left(\lambda \right)$ approaches a zero matrix which corresponds to an empty network without any edges; hence, *Dens*(λ_0_) is close to zero. As λ_*n*_ decreases, ${\hat{\Omega }}_{*}\left(\lambda \right)$ becomes denser and more elements become non-zero, resulting in the increase in *Dens*(λ_*n*_). As *n* increases and λ_*n*_ → 0, *Dens*(λ_*n*_) reaches a plateau and becomes stabilized with further decrease in λ_*n*_. With a finite sequence of {λ_*n*_} we can find the maximum of *Dens*(λ_*n*_), and denote it as *Dens*_*max*_. In practice, it is not necessary to select λ_*max*_ that corresponds to *Dens*_*max*_, because it is somewhat arbitrary and depends on the smallest value specified in the finite sequence of {λ_*n*_}. Instead, based on the profile of *Dens*(λ_*n*_), users can choose the value in the sequence that corresponds to the plateau point in the profile, which is denoted as $\lambda_{\text{platu}}^{*}$. After $\lambda_{\text{platu}}^{*}$, *Dens*(λ) becomes stabilized and only increases by a trivial amount when further decreasing the tuning parameter. Specifically, we define $\lambda_{\text{platu}}^{*}$ as the largest λ_*n*_ in the sequence such that for any λ_k_ ≤ λ_*n*_, we have

$$\begin{array}{l} \frac{\left|Dens\left(\lambda_{\text{k}}\right)-{Dens}_{\text{max}}\right|}{{Dens}_{\text{max}}}\leq \varepsilon , \end{array}$$

where ε is a user-specified small value such as 0.01. Since the estimated network is close to the maximum density level at $\lambda_{\text{platu}}^{*}$, ${\hat{\Omega }}_{*}\left(\lambda_{\text{platu}}^{*}\right)$ corresponds to the estimate of the precision matrix that is obtained under minimum sparsity constraint.

As the number of nodes in the network increases, it may be desirable to impose a certain sparsity regularization to reduce the number of false positive edges in the estimated precision matrix. In this case, we propose the following method to select the tuning parameter based on a user-specified *Dens* level for the precision matrix estimate. Suppose the user would like to obtain an precision matrix estimate that would reach *p* percent of the maximum density level, that is *Dens*(λ_*n*_)=*p*×*Dens*_*max*_, then the corresponding tuning parameter $\lambda_{p}^{*}$ can be selected from {λ_*n*_} as follows:

(6) $$\begin{array}{l} \lambda_{p}^{*}={\text{argmin}}_{\lambda_{\text{n}}}\left\{\left|Dens\left(\lambda_{\text{n}}\right)-p\times {Dens}_{\text{max}}\right|\right\}. \end{array}$$

After we select the tuning parameter and obtain the CLIME estimate Ω^*^ of the precision matrix, we can derive the partial correlation matrix estimate, ***Pcorr*** = {ρ_ij_}MxM, via the following equation:

(7) $$\begin{array}{l} \text{Pcorr}=-\text{diag}{\left(\Omega \right)}^{-1∕2}\Omega \text{diag}{\left(\Omega \right)}^{-1∕2}+2{\text{I}}_{\text{M}}. \end{array}$$

In summary, we have proposed a novel tuning parameter selection criterion for the sparse precision matrix estimation in brain network modeling. A detailed summary of the steps of our procedure is provided in Table 1.

**Table 1:** **Proposed *Dens*-based partial correlation estimation approach**.

**Summary steps:**
**Input:** Estimate the sample covariance matrix $\hat{\Sigma }$ based on the observed fMRI time series from M nodes in the brain. If one would like to impose sparsity regularization on the precision matrix estimate, specify a percentage *p*, where *p* ∈ (0, 1), for selecting the tuning parameter based on the desired density level of the precision matrix estimate. **Step 1**, **Select the Tuning Parameter** •Specify a monotonically decreasing sequence {λ_*n*_, *n* = 0, 1, …,} within the range (0,1) with λ_0_ → 1 and λ_1_ → 0 as n increases. •Obtain CLIME estimate based on each value in {λ_*n*_} starting from λ_0_. Keep decreasing λ_*n*_ until *Dens*(λ_*n*_) reaches its plateau and remains stable afterwards. •Denote the maximum *Dens*(λ_*n*_) in its profile as *Dens*_max_ and the tuning parameter that corresponds to the plateau point in the profile as λ_platu_. •If the user specifies a percentage *p* that represents the desired *Dens* level, find the corresponding tuning parameter λ^*^_p_ from the sequence {λ_n_} based on (4). **Step 2, Estimate the precision matrix using CLIME** Based on the selected tuning parameter λ, obtain CLIME estimate ${\hat{\Omega }}_{*}\left(\lambda \right)$ through the procedure in (3) and (4) **Step3, Derive estimate for the partial correlation matrix** Obtain $\hat{Pcorr}$ from ${\hat{\Omega }}_{*}\left(\lambda \right)$ using Equation (7)

## Simulation studies and results

In this section, we investigate the empirical performance of the proposed tuning parameter selection method using synthetic data. We simulated spatially- and temporally-dependent data that mimic real fMRI data. Specifically, to induce spatial dependence between the nodes, we generated data from specified networks and considered various sparsity levels for the network. We then evaluated the performance of the proposed tuning parameter selection method based on the *Dens* criterion and compared that to the existing selection methods.

### Synthetic data

We generated time series data for M nodes over T time points. Real fMRI data, which are collected over a series of time points, demonstrate both temporal and spatial dependence. In order to mimic this complex covariance structure, we first specified a precision matrix **Ω** that represent the network connectivity among the M nodes, the spatial covariance matrix **Σ**_s_ can be derived from **Ω**. We then induced temporal correlation in the node time series via an AR(1) model. The detailed procedure is presented as follows. Let **Y** be the TxM data matrix. Based on a pre-specified precision matrix **Ω**, **Y**_**Ω**_ were generated as:

(8) $$Y_{\Omega }=X+Z$$

where $X={\left\{X_{1},\ldots ,X_{M}\right\}}^{\text{T}}$ was a TxM matrix where each row $X^{\prime }{\;}_{i}\text{s}\sim_{\text{iid}}{\text{N}}_{\text{M}}\left(0,\Sigma_{\text{s}}\right)$. Here $\Sigma_{\text{s}}=\Omega^{-1}-{\tau^{2}\text{I}}_{\text{M}}$ is the spatial covariance matrix derived from **Ω**. **Z** = {**Z**_1_, …, **Z**_**M**_} was also a TxM matrix where each column ${\text{Z}}_{\text{i}}^{\prime }\text{s}\sim_{\text{iid}}{\text{N}}_{\text{T}}\left(0,\Sigma_{\text{T}}\right)$ with $\Sigma_{\text{T}}=\left\{\Sigma_{\text{T},\text{ij}}\right\}=\left\{\tau^{2}\gamma^{\left|\text{i}-\text{j}\right|}\right\}$ being the temporal covariance matrix based on an AR(1) model.

In the data generation model (8), **X** induces the spatial covariance structure in the data which is controlled by the precision matrix, and **Z** induces the temporal correlations in the data which are AR(1) time series with variance τ^2^ and adjacent correlation γ. In order to ensure that the spatial covariance matrix **Σ**_s_ is positive definite, the variance τ^2^ is set to be half of the inversed largest eigenvalue of **Ω**. As a result, **Y**_**Ω**_ generated from (8) has a matrix normal distribution and the precision matrix of **Y**_**Ω**_ in the spatial domain is **Ω**.

In our simulation, we generated data from (8) with M = 10 and T = 50. To examine the performance of the proposed *Dens* criterion under various scenarios, we considered 9 sparsity levels ranging from 0.29 to 0.93, where the sparsity level represents the percentage of non-zero elements in the off-diagonal. For each scenario, we had 100 simulation runs.

In the next section, we evaluated the performance of the proposed *Dens*-based regularization selection method, and compared to four existing selection methods including the AIC, BIC, and K-CV approaches with the negative log likelihood and trace L2 loss functions. For our proposed *Dens*-based selection method, we selected three tuning parameters corresponding to different density levels: $\lambda_{\text{platu}}^{*}$ which leads to an estimate which corresponds to the plateau point in the *Dens* profile, and $\lambda_{0.45}^{*}$ and $\lambda_{0.75}^{*}$ which lead to estimates that reach 45 and 75% of the maximum density level, respectively. For K-CV methods, we used 5-fold cross validation for selecting λ.

To evaluate the performance of the various methods in estimating the partial correlation matrix, we calculated the MSE, sensitivity, and specificity by comparing the true and estimated partial correlations from different methods. Here, the MSE is obtained as the average MSE across all off-diagonal edges in the partial correlation matrix.

### Results from the simulation study

We present detailed simulation results for each of the 9 sparsity levels in Tables 2–4. We also present the average results across all sparsity levels and as well as the average computation time for these methods in Tables 5, 6.

**Table 2:** **Comparison of MSE for estimated partial correlation matrix based on different regularization selection methods across various sparsity levels with the simulated data**.

**Methods**	**Sparsity level**								
	**0.29**	**0.36**	**0.47**	**0.49**	**0.56**	**0.76**	**0.87**	**0.89**	**0.93**
K-CV log like	0.016	0.031	0.026	0.014	0.015	0.014	0.019	0.011	0.010
K-CV Trace L2	0.012	0.018	0.017	0.009	0.010	0.011	**0.011**	0.011	0.011
AIC	0.016	0.031	0.026	0.014	0.015	0.014	0.019	0.011	0.010
BIC	0.016	0.031	0.026	0.014	0.015	0.014	0.019	0.011	0.010
λ*_0.45_	**0.010**	**0.015**	**0.012**	**0.006**	0.010	0.012	**0.011**	0.015	0.016
λ*_0.75_	**0.010**	0.020	0.017	0.008	**0.009**	**0.009**	0.012	**0.009**	**0.008**
λ*_platu_	0.014	0.027	0.023	0.012	0.012	0.012	0.016	0.010	0.009

Based on simulated data, we examined our proposed Dens method and commonly used regularization selection methods including K-CV with negative log likelihood, Trace L2, AIC, BIC. For Dens method, we adopt three different density level: 45, 75% and plateau, corresponding to λ*_0.45_, λ*_0.75_, and λ*_platu_ separately. The MSE values in bold are the optimal result across the different methods at each sparsity level.

**Table 3:** **Comparison of Sensitivity for identifying connections based on different regularization selection methods across various sparsity levels with the simulated data**.

**Methods**	**Sparsity level**								
	**0.29**	**0.36**	**0.47**	**0.49**	**0.56**	**0.76**	**0.87**	**0.89**	**0.93**
K-CV log like	1.00	1.00	1.00	1.00	1.00	1.00	1.00	1.00	1.00
K-CV Trace L2	0.95	0.86	0.81	0.86	0.85	0.83	0.81	0.86	0.84
AIC	1.00	1.00	1.00	1.00	1.00	1.00	1.00	1.00	1.00
BIC	1.00	1.00	1.00	1.00	1.00	1.00	1.00	1.00	1.00
λ*_0.45_	0.85	0.79	0.63	0.74	0.67	0.65	0.62	0.57	0.61
λ*_0.75_	0.96	0.92	0.86	0.93	0.87	0.91	0.90	0.91	0.93
λ*_platu_	0.99	0.97	0.96	0.98	0.97	0.98	0.97	0.99	0.99

**Table 4:** **Comparison of Specificity for identifying connections based on different regularization selection methods across various sparsity levels with the simulated data**.

**Methods**	**Sparsity level**								
	**0.29**	**0.36**	**0.47**	**0.49**	**0.56**	**0.76**	**0.87**	**0.89**	**0.93**
K-CV log like	0.00	0.00	0.00	0.00	0.00	0.00	0.00	0.00	0.00
K-CV Trace L2	0.22	0.35	0.30	0.39	0.29	0.37	0.40	0.28	0.41
AIC	0.00	0.00	0.00	0.00	0.00	0.00	0.00	0.00	0.00
BIC	0.00	0.00	0.00	0.00	0.00	0.00	0.00	0.00	0.00
λ*_0.45_	0.75	0.61	0.64	0.77	0.72	0.73	0.75	0.81	0.82
λ*_0.75_	0.20	0.13	0.16	0.19	0.25	0.21	0.23	0.22	0.24
λ*_platu_	0.04	0.03	0.04	0.04	0.05	0.04	0.05	0.04	0.05

**Table 5:** **Averaged performance of regularization methods across various sparsity levels with the simulated data**.

**Methods**	**MSE**	**Sensitivity**	**Specificity**	**Average of Sens. and Spec**.	**Computation time (Secs)**
K-CV log like	0.017	1.000	0.000	0.500	2.7268
K-CV Trace L2	0.012	0.853	0.333	0.593	2.7079
AIC	0.017	1.000	0.000	0.500	0.0027
BIC	0.017	1.000	0.000	0.500	0.0016
λ*_0.45_	0.012	0.680	0.731	**0.706**	**0.0004**
λ*_0.75_	0.011	0.910	0.204	0.557	**0.0004**
λ*_platu_	0.015	0.978	0.041	0.509	**0.0004**

The values in bold are the optimal result across the different methods.

**Table 6:** **Comparison of computational time to select the tuning parameter for one randomly selected subject from the PNC study using different regularization selection methods**.

**Methods**	**K-CV log like**	**K-CV TraceL2**	**AIC**	**BIC**	λ*_0.45_	λ*_0.75_	λ*_platu_
Computational time (Secs)	8575.93	8257.72	0.242	0.229	**0.004**	**0.004**	**0.004**

The values in bold are the optimal result across the different methods.

Compared with the existing methods, the proposed *Dens*-based method is much more computationally efficient, especially compared to the K-CV methods (Tables 5, 6). The computational efficiency provides an important advantage in estimating brain networks based on high-dimensional fMRI data. In addition, our proposed method provided the most accurate estimation in terms of the average MSE and the number of times it achieved the lowest MSE value across different sparsity levels (see Tables 2, 5). This indicates that our method has better accuracy, on average, across different sparsity levels. In terms of sensitivity and specificity, AIC, BIC, and K-CV with a negative log likelihood loss function tended to select an overly dense network with extremely low specificity, which was consistent with previous findings in the literature. In comparison, K-CV based on Trace L2 loss function provided more balanced performance in terms of sensitivity and specificity. For our method, $\lambda_{\text{platu}}^{*}$, also tended to select an overly dense network, which is expected since it imposes the minimum sparsity regularization. For the $\lambda_{0.45}^{*}$ and $\lambda_{0.75}^{*}$ which applied sparsity constraints, we achieved much better balance between sensitivity and specificity. In particular, $\lambda_{0.45}^{*}$ offers the best average of sensitivity and specificity at 0.706, which is much higher than those of the four existing methods (see Table 5). In summary, our proposed *Dens*-based method provided comparable or better performance with respect to the existing methods but only used a small fraction of computation time required by the other methods (see Table 6). Furthermore, the *Dens*-based method provides investigators an intuitive and flexible way to select the tuning parameter according to desired density level they would like to impose on the network estimates.

## Application to rs-fMRI data from the philadelphia neurodevelopmental cohort (PNC)

### PNC study and description

The PNC is a collaborative project between the Brain Behavior Laboratory at the University of Pennsylvania and the Children's Hospital of Philadelphia (CHOP), funded by NIMH through the American Recovery and Reinvestment Act of 2009 (Satterthwaite et al., 2014, 2015). The PNC study includes a population-based sample of over 9500 individuals aged 8–21 years selected among those who received medical care at the Children's Hospital of Philadelphia network in the greater Philadelphia area; the sample is stratified by sex, age and ethnicity. A subset of participants from the PNC were recruited for a multimodality neuroimaging study which included resting-state fMRI (rs-fMRI). In this paper, we considered rs-fMRI data from 881 participants in the PNC study that were released in the dbGaP database. Compared to many other large-scale publicly available rs-fMRI datasets, the PNC data has a major advantage that all the images were acquired on a single MRI scanner using the same scanning protocol. Hence, the images from the PNC data do not suffer from extra variation caused by different scanners or protocols.

All images from the PNC study were acquired on a Siemens Tim Trio 3 Tesla, Erlangen, Germany using the same imaging sequences. The rs-fMRI scans were acquired with 124 volumes, TR 3000ms, TE 32 ms, flip angle 90°, FOV 192 × 192 mm, matrix 64 × 64 and effective voxel resolution 3.0 × 3.0 × 3.0 mm. More details about experimental settings and image acquisition can be found in Satterthwaite et al. (2015).

Prior to analysis, we performed a quality control procedure on the rs-fMRI. Specifically, we removed subjects who had more than 20 volumes with relative displacement >0.25 mm to avoid images with excessive motion (Satterthwaite et al., 2015). Among the 881 subjects who had rs-fMRI scans, 515 participants' data met the inclusion criterion and were used in our following analysis. Among these 515 subjects, 290 (56%) were female and the mean age was 14.51 years (SD = 3.32).

### Rs-fMRI data preprocessing

The rs-fMRI data were preprocessed using the preprocessing script released from the 1000 Functional Connectomes Project. Specifically, skull stripping was performed on the T1 images to remove extra-cranial material, then the first four volumes of the functional time series were removed to stabilize the signal, leaving 120 volumes for subsequent preprocessing. The anatomical image was registered to the 8th volume of the functional image and subsequently spatially normalized to the MNI standard brain space. These normalization parameters from MNI space were used for the functional images, which were smoothed with a 6 mm FWHM Gaussian kernel. Motion corrections were applied on the functional images. A validated confound regression procedure (Satterthwaite et al., 2015) was performed on each subject's time series data to remove confounding factors including motions, global effects, white matter (WM) and cerebrospinal fluid (CSF) nuisance signals. The confound regression contained nine standard confounding signals (6 motion parameters plus global/WM/CSF) as well as the temporal derivative, quadratic term and temporal derivative of the quadratic of each. Furthermore, motion-related spike regressors were included to bound the observed displacement. Lastly, the functional time series data were band-pass filtered to retain frequencies between 0.01 and 0.1 Hz which is the relevant frequency range for rs-fMRI.

### Brain network construction

**Figure 2:**
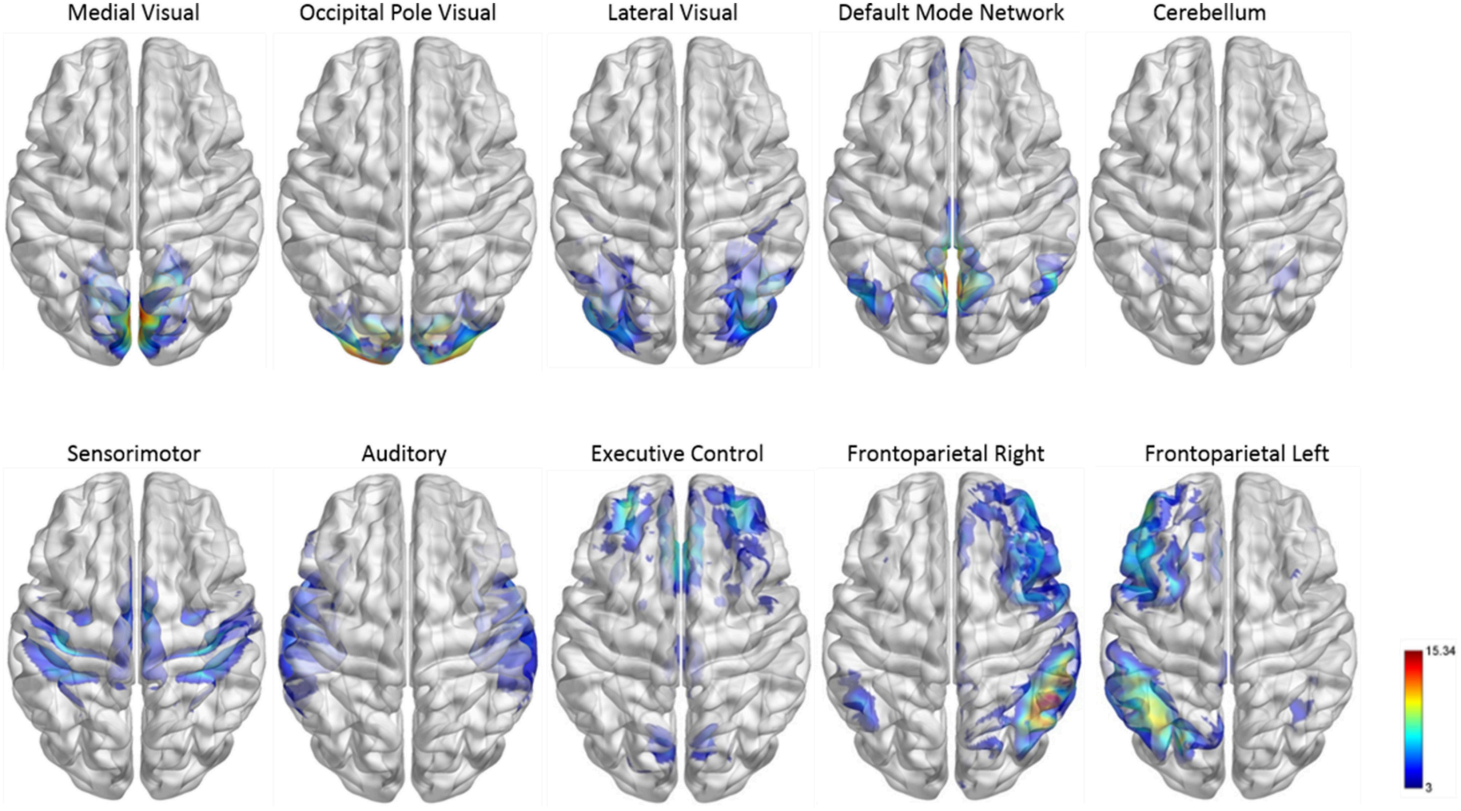
**Functional module maps**. The functional module z-score maps (thresholded at z > 3) defined by the 10 primary resting state networks (RSNs, Smith et al., 2009). To categorize nodes by module membership, we find the RSN map with the largest z-score in the location of the node, above a certain threshold (z > 3).

In fMRI, brain activity is measured at voxel level, which are regions a few cubic millimeters in size. A typical 3D fMRI scan contains hundreds of thousands of voxels across the brain. The first step in brain network construction is usually to select a set of network nodes across the brain. Using individual voxels as network nodes has several issues: it results in an extremely high-dimensional connectivity matrix that is computationally challenging to estimate, and the voxel-based network tends to be very noisy due to the high noise level of fMRI BOLD signals in individual voxels. Additionally, a voxel-based network is highly variable across subjects due to the difficulty of matching different subjects' brains at the voxel level. On the other hand, defining nodes by a coarse parcellation of the brain into large functionally homogenous regions can cause a loss in spatial resolutions when investigating the connectivity between brain locations. In our paper, we adopted the 264-node cortical parcellation system defined by Power et al. (2011). This system of nodes was determined using a combination of meta-analysis of task-based fMRI studies and resting state functional connectivity mapping techniques. In this network, each node is a 10 mm diameter sphere in standard MNI space representing a putative functional area, and the collection of nodes provides good coverage of the whole brain (see Figure 2). This node system provides a good balance of spatial resolution and dimension reduction. It is a finer spatial resolution than the commonly used Automated Anatomical Labeling (AAL) atlas (Tzourio-Mazoyer et al., 2002), but is not as granular as using a system of single voxels. This kind of intermediate node scheme is recommended to balance the trade-off between increased spatial resolution and attenuate signal-to-noise ratio (Fornito et al., 2010; Power et al., 2011).

**Figure 3:**
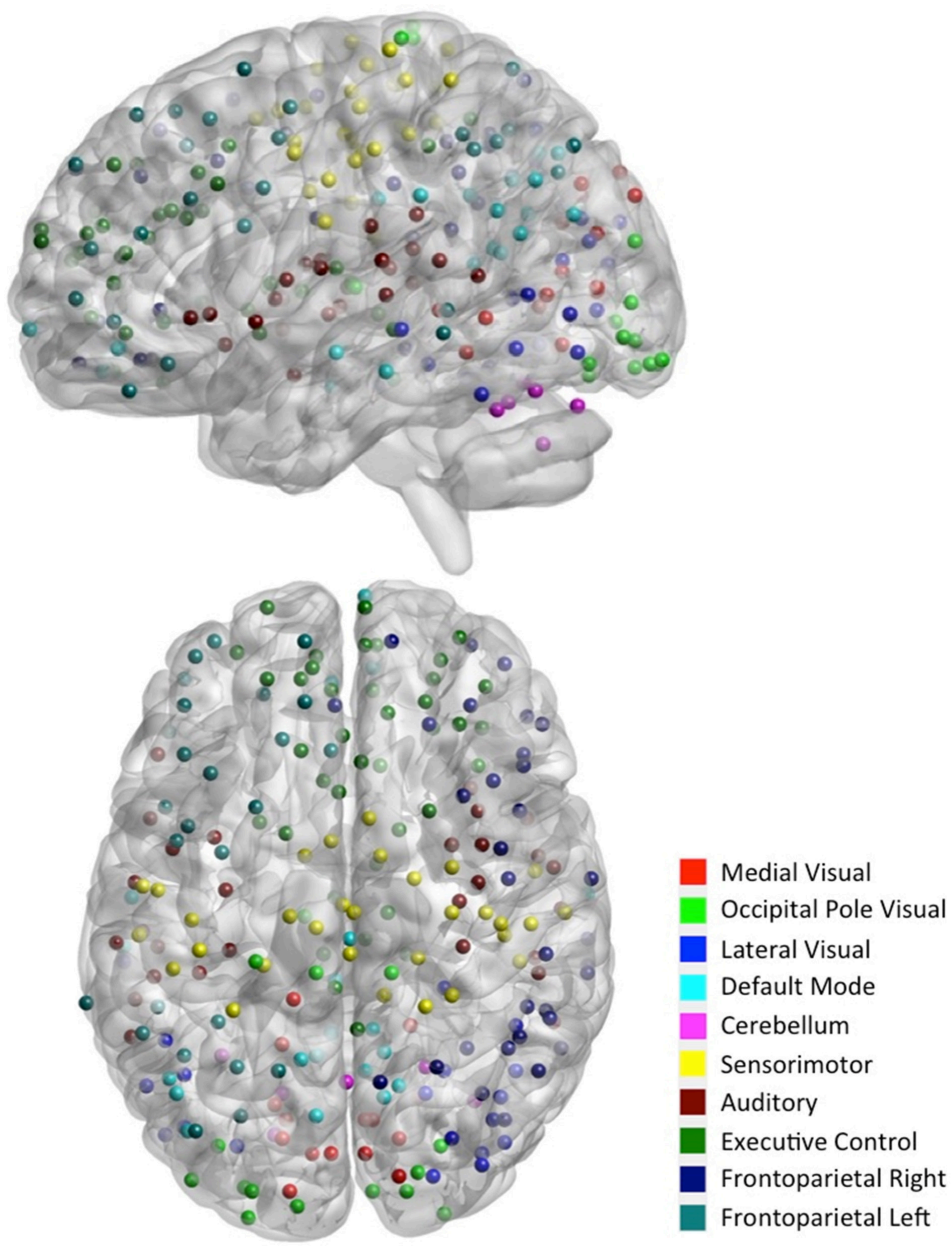
**Parcellation scheme and network assignment**. The 232 nodes used in our network analysis are adapted from the 264-node parcellation system (Power et al., 2011). Each node is a 10 mm diameter sphere in MNI space representing a putative functional area, and is color-coded to indicate its module membership. Functional modules are defined by 10 primary RSNs (Smith et al., 2009).

To facilitate the understanding of the functional roles of the nodes, we assigned them to 10 functional networks or “modules” that correspond to the major resting state networks (RSNs) described by Smith et al. (2009) (see Figure 2). The RSN maps, determined by ICA decomposition of a large database of activation studies (BrainMap) and rs-fMRI data, are coherent during both task activity and at rest. The functional modules include medial visual network (“Med Vis,” 15 nodes), occipital pole visual network (“OP Vis,” 15 nodes), lateral visual network (“Lat Vis,” 19 nodes), default mode network (“DMN,” 20 nodes), cerebellum (“CB,” 6 nodes), sensorimotor network (“SM,” 31 nodes), auditory network (“Aud,” 29 nodes), executive control network (“EC,” 39 nodes), and right and left frontoparietal networks (“FPR” and “FPL,” 32 and 26 nodes, respectively). To determine the module membership at each node, we found the RSN map with the largest z-value in the location of the node, above a certain threshold (z > 3). Thirty two of the 264 nodes were not strongly associated with any RSN maps, and were therefore not included. A visualization of the remaining 232 nodes, classified by functional module, is shown in Figure 3. All brain visualizations were created using BrainNet Viewer (Xia et al., 2013).

To construct the network, we extracted the time series from each node with the following steps. First, the time series at each voxel were detrended, demeaned, and whitened. We then averaged the time series for all the voxels in each node to represent the node-specific time series. These node-specific time series were then used in subsequent analyses to estimate connectivity in the network. We note that using the within-node average or SVD time series in network construction is only appropriate when such summarized time series sufficiently represent the temporal dynamics within each node. When one uses a coarse brain parcellation such as the AAL regions in network construction, such this dimension reduction can cause problems in accurate estimation of the conditional independence structure in a network (Han et al., 2014). In the next section, we describe the estimation of the 232 × 232 connectivity matrix using partial correlation to measure direct brain connectivity. For comparison, we also estimated a connectivity matrix based on full correlation for each subject to examine marginal connectivity between the nodes.

### Estimation of the partial correlation matrix

We applied the proposed method to estimate the partial correlation matrix based on the rs-fMRI data from the PNC study. For a given subject, we first obtained the sample covariance matrix based on the time series from each node. We then estimated the precision matrix from the sample covariance matrix using the CLIME method.

#### Comparison between *Dens*-based method and existing methods for selecting the tuning parameter

**Figure 4:**
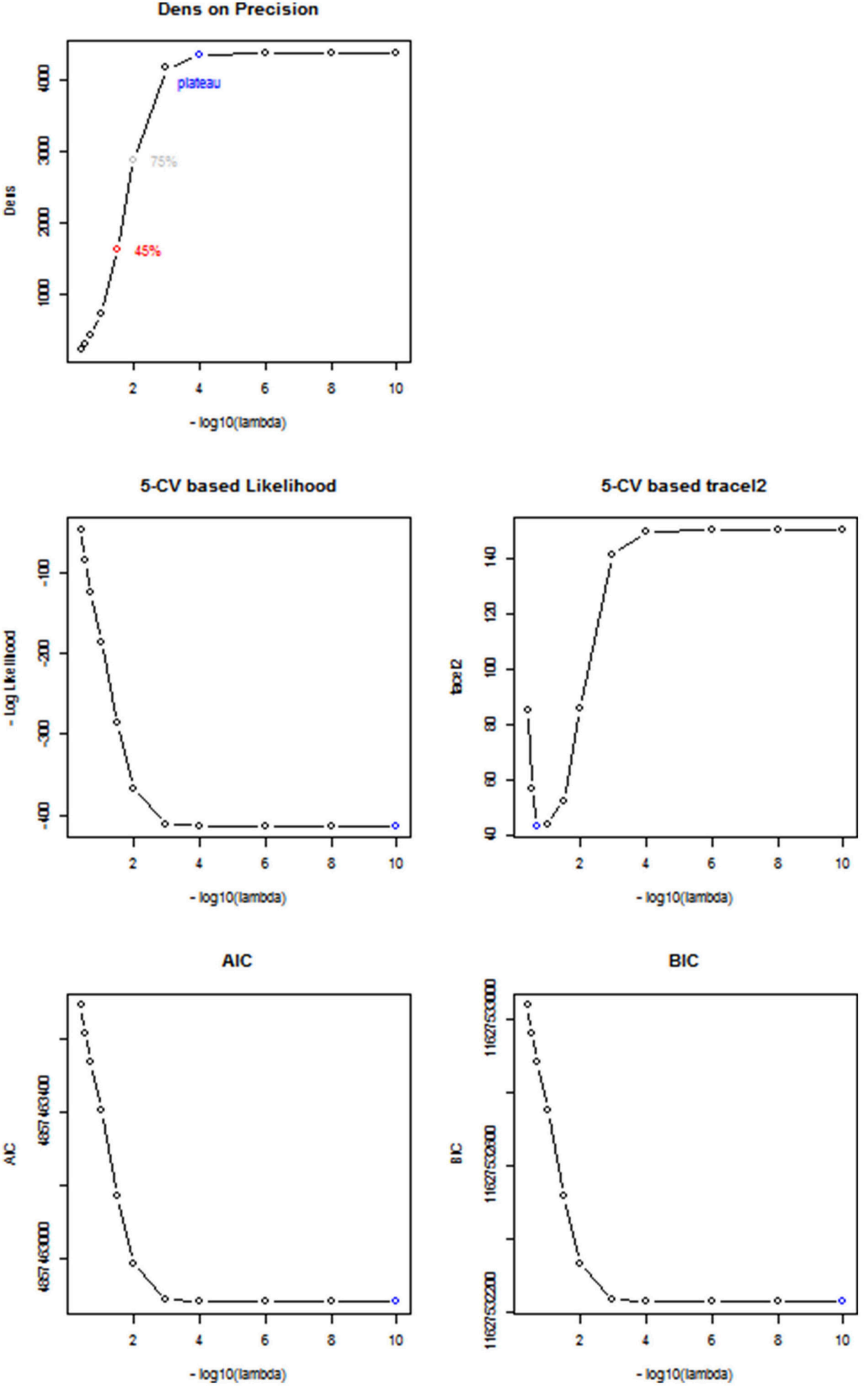
**Comparison between different Regularization methods**. Based on a randomly selected subject, we compared the performance between 5 different regularization methods including *Dens* method, 5-CV based negative log likelihood, 5-CV based TraceL2, AIC, and BIC, where λ values are on the −log_10_ scale, ranging from 10^−10^ to 0.4. The selected λ under each method is in blue.

To choose the tuning parameter for CLIME, we applied the proposed method based on the *Dens* criterion and considered $\lambda_{\text{platu}}^{*}$, $\lambda_{0.45}^{*}$ and $\lambda_{0.75}^{*}$. In comparison, we also considered other existing methods including AIC, BIC, and a 5-fold K-CV approach with the negative log likelihood and Trace L2 loss functions. In Figure 4, we plotted the profiles of the objective functions adopted by these methods for choosing the tuning parameter across a series of λ values, ranging from 1e-10 to 0.4, for a randomly selected subject in the PNC study. In Table 5, we present the selected tuning parameter and the associated computation time based on each of these methods. From Figure 4, we can see the profile of the objective function based on *Dens* shows a similar pattern with the profiles of the AIC, BIC and the negative log likelihood 5-CV. All four of these profiles show that the objective functions improve significantly when λ was decreased from 0.4 to 1e-3, reach a plateau around 1e-4, and then only had very small changes when λ was further decreased. However, since these three existing methods (negative log likelihood 5-CV, AIC, BIC) all choose the λ that maximizes their corresponding objective function, they ended up choosing the minimum λ, i.e., 1e-10, in the series. In contrast, the Trace L2-based 5-CV method had a different pattern which selected a value of λ = 0.2. This corresponds to a fairly strong sparsity constraint in CLIME and leads to the sparsest estimate of the partial correlation matrix among all these methods.

Our proposed *Dens*-based selection method was the most efficient of all the methods considered. In particular, it showed a dramatic reduction in computation time as compared with the cross-validation methods. Unlike the AIC, BIC and negative log likelihood-based 5-CV methods which always selected the minimum λ = 1e-10, our proposed *Dens*-method was much more flexible in terms of selecting tuning parameters that correspond to various density levels that users may be interested in. Specifically, we found that the network corresponding to $\lambda_{\text{platu}}^{*}$ = 1e-4 was extremely close to the estimated network based on λ = 1e-10 chosen by the AIC, BIC and negative log likelihood-based methods. We found that $\lambda_{0.45}^{*}=0.032$ and it induced less stringent sparsity control as compared to λ = 0.2 as selected by the Trace L2 method. For λ = 0.2, we can see from Figure 4 that it only reaches 10% of the *Dens* level in the unconstrained estimates of the network.

**Figure 5:**
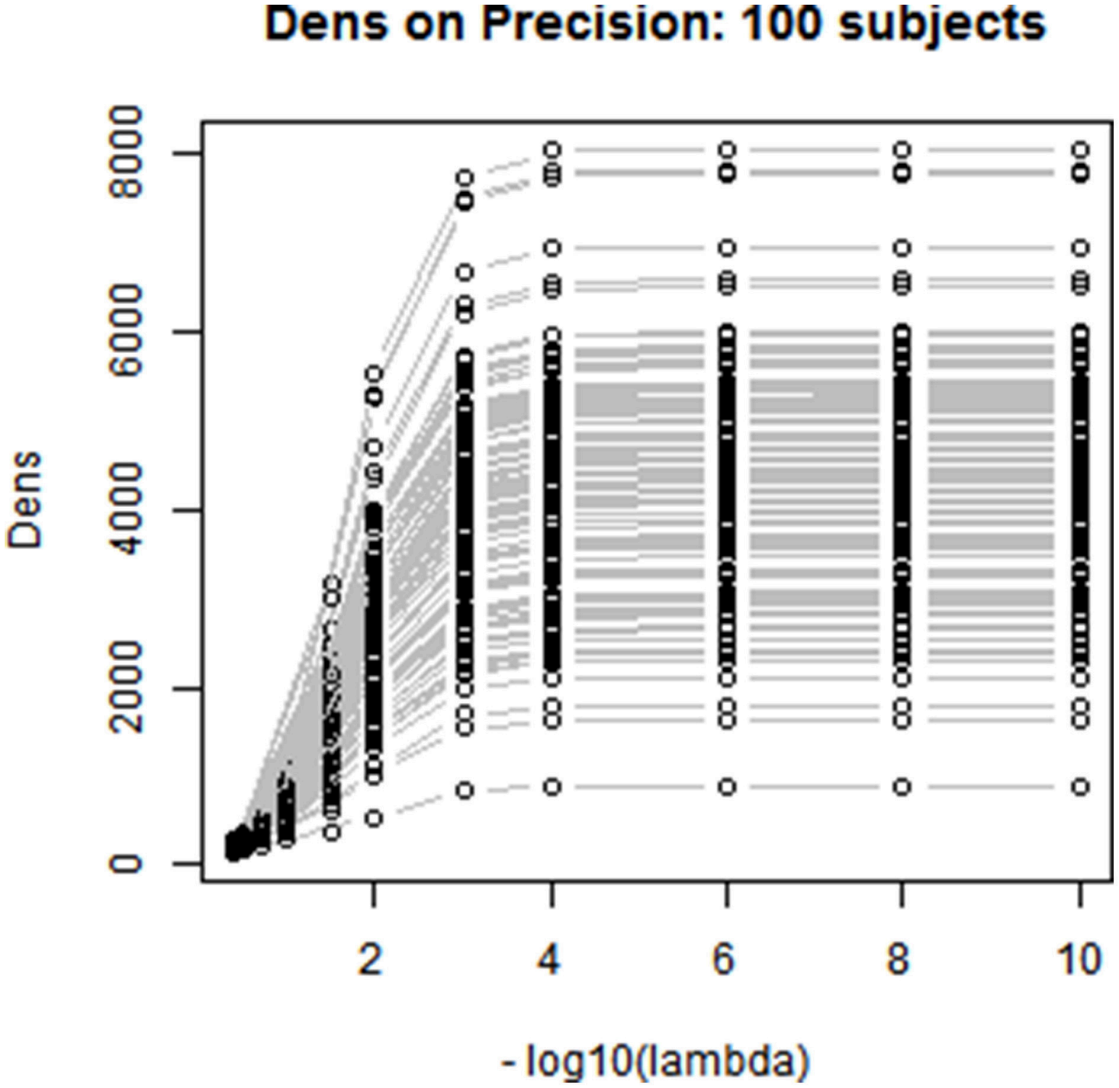
**Between-subjects consistency analysis for the proposed *Dens-based* method**. We randomly selected 100 subjects to check the consistency of the proposed *Dens* method, where λ values are on the −log_10_ scale, ranging from 1e-10 to 0.4. As shown in the figure, *Dens* method is highly consistent across subjects serving as a reliable property to select the tuning parameters for large group level study.

We also investigated the consistency of the results based on the *Dens*-based selection method across subjects. We randomly selected 100 subjects from the PNC study and applied the proposed method for choosing the CLIME tuning parameter for estimating subject-specific precision matrices. Figure 5 displays the profiles of the *Dens* objective function across subjects. The results show that the proposed *Dens* objective function demonstrates a consistent pattern across subjects, and we also found consistent values across all 100 subjects for $\lambda_{\text{platu}}^{*}$, $\lambda_{0.45}^{*}$, and $\lambda_{0.75}^{*}$. Based on this finding, it is well-justified for us to apply the same tuning parameter to estimate partial correlation matrices for all subjects in the PNC study. This greatly facilitates between-subject comparisons and also allows the construction of a group-level partial correlation matrix by combing subject-specific estimates.

#### Comparison between the proposed method and existing methods for estimating partial correlation

**Figure 6:**
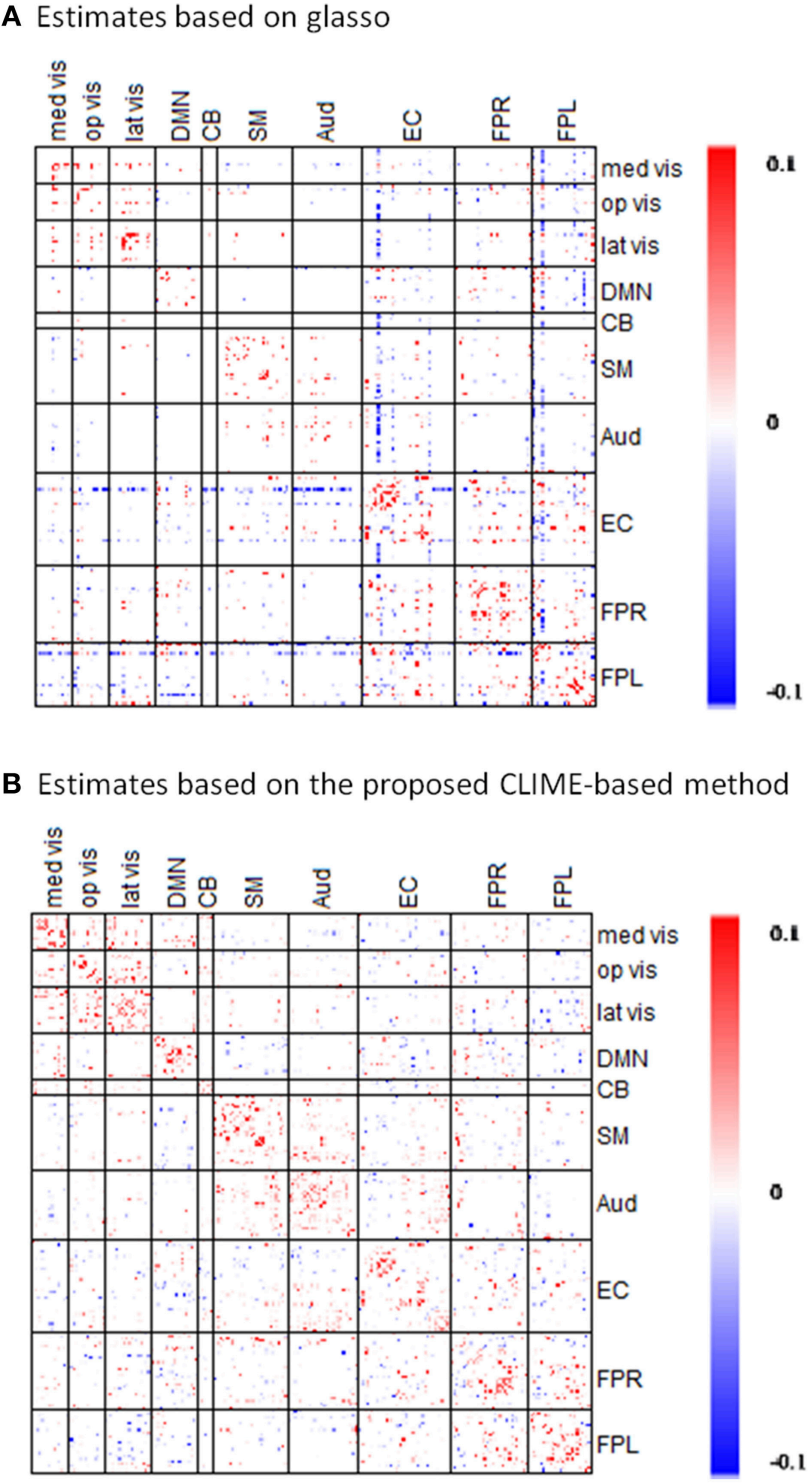
**Comparisons of partial correlation estimates based on the proposed CLIME-based method and based on GLASSO method**. The symmetric 232 × 232 partial correlation matrices are estimated via the **(A)** existing GLASSO approach and the **(B)** proposed CLIME-based approach using rs-fMRI data from a randomly selected subject in the PNC study. Sparsity regularization was set at similar level in both methods. Red indicates the positive edges and blue indicates the negative edges.

Using the fMRI data from the PNC study, we compared the performance of the proposed *Dens*-based method with two existing methods for estimating partial correlation. We first compared to the method referred to as the L1 precision method (Schmidt, 2006) which was used to obtain partial correlation in the well-known network modeling paper by Smith et al. (2011). The L1 precision method requires selection of a regularization-controlling parameter λ^#^. We considered the values within the range used in Smith et al. (2011) which includes λ^#^ = 1 and 5. For these regularization values, the L1 precision method produced a diagonal matrix, which is an overly sparse estimate for the precision matrix. To fix this issue, we decreased λ^#^ to 0.1 and 0.5 to obtain a less sparse precision matrix. However, in these cases, the L1 precision algorithm failed to provide valid estimates and produced precision matrices with complex values. Furthermore, the L1-precision method is much more time-consuming than the proposed approach, using 1573 s for estimating a single subject's precision matrix at λ^#^ = 5. When we specified λ^#^ = 0.1 in order to obtain a less sparse precision matrix, the computation time dramatically increased to 12,849 s per subject. In comparison, our proposed method produced valid estimates of the partial correlation matrix for all 515 subjects in PNC data. Our method was also significantly faster than the L1 precision method, only taking about 58–60 s per subject. In addition, we also considered another existing method for estimating partial correlation based on the glasso R package (Schmittmann et al., 2015). When comparing the results (Figure 6), one major distinction is that connectivity matrix based on our proposed method showed more positive connections within-modules nodes suggesting within-module are more densely connected to one another than to the rest of the network. In comparison, glasso-based connectivity matrix showed less within-module positive connections and in some cases even produced strong negative connections within the same functional module. Based on the network comparison criterion in the literature (Power et al., 2011), these results suggest that the connectivity matrix based on our proposed method more accurately reflects the brain organization in the sense that it better captures the strong positive functional connections within the established functional modules.

### Comparison of network connectivity based on partial correlation and full correlation

**Figure 7:**
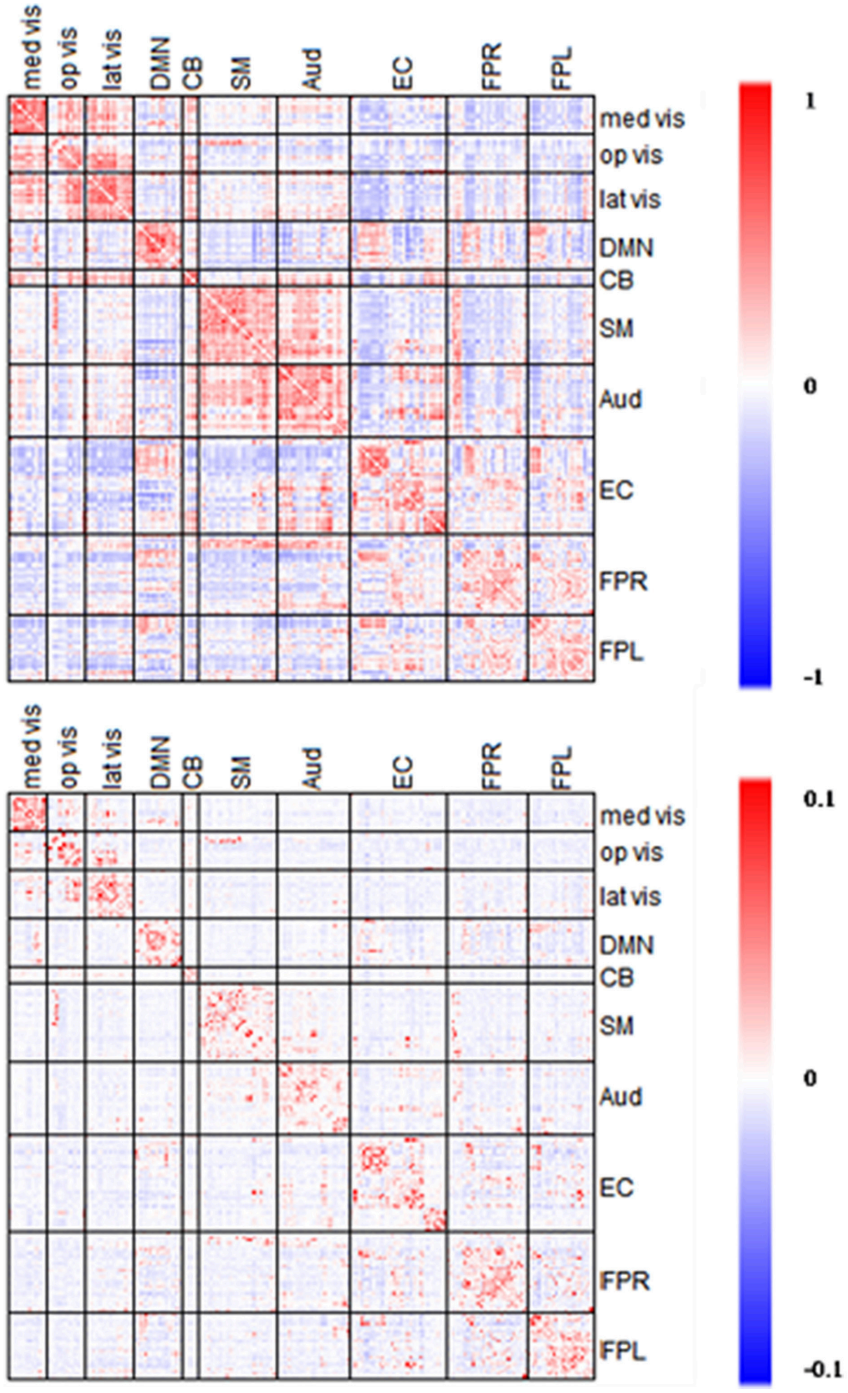
Averaged edgewise partial correlation matrix (λ^*^_platu_) and full correlation matrix. The symmetric 232 × 232 connectivity matrices under partial correlation (λ^*^_platu_, **bottom**) and full correlation (Pearson correlation, **top**). Red indicates the positive edges and blue indicates the negative edges.

In the section, we compare the partial-correlation-based network connectivity and full correlation-based connectivity for the PNC study. Following the method from Satterthwaite et al. (2014), we did not threshold the correlation matrix, yielding a fully connected correlation matrix. Thus, to ensure comparability, we imposed minimum sparsity control in the partial correlation estimation and selected $\lambda_{\text{platu}}^{*}$ for the CLIME. Figure 7 displays the partial correlation matrix and correlation matrix averaged across the 515 subjects in PNC data.

Full correlation values ranged between −0.45 and 0.83, and in comparison, partial correlation values ranged between −0.03 and 0.18. As expected, the magnitude of partial correlation was much smaller than full correlation since the partial correlation reflects the direct connections between nodes after removing the confounding effects from all the other nodes. Based on the 10 functional module system defined by Smith et al. (2009), we divided the upper-triangle of the 232 × 232 edgewise connectivity matrices into 55 module-wise blocks including the 10 within-module blocks and 45 between-module blocks. In the full correlation-based connectivity matrix, we can see the majority of positive marginal connections were found in within-module blocks, that is the diagonal blocks in the connectivity matrix. We also found positive connections in several between-module blocks, in particular between the three visual networks (Med Vis, Op Vis, Lat Vis) and also between the Auditory (Aud) and Sensorimotor (SM) network. In the partial correlation-based connectivity matrix, the strong positive connection in within-module blocks became even more prominent, indicating that the most significant positive direct connections in the brain are observed within functional modules, and for between-module node pairs we observed fewer positive connections as compared to the full correlation matrix. For example, we observed fewer positive connections between the Auditory (Aud) and Sensorimotor (SM) network. Similarly, the connections between the three visual networks had dropped considerably too as compared to the full correlation matrix. These findings suggest that a lot of the marginal connections for between-module node pairs are mainly due to some confounding factors and not necessarily due to the direct connections between modules. Another important finding is that in the full correlation-based connectivity matrix, there were considerable negative functional connections in the between-module blocks. Several of these negative marginal connections disappeared in the partial correlation matrix, indicating that many of the negative connections may be caused by confounding factors. This finding agrees with some recent findings in the neuroimaging community that showed many negative functional connections in rs-fMRI may be due to non-neurological reasons such as global signal removal performed during imaging pre-processing (Giove et al., 2009; Murphy et al., 2009; Weissenbacher et al., 2009; Chen et al., 2011) or inhomogeneous cerebral circulation across the brain (Goelman et al., 2014).

**Figure 8:**
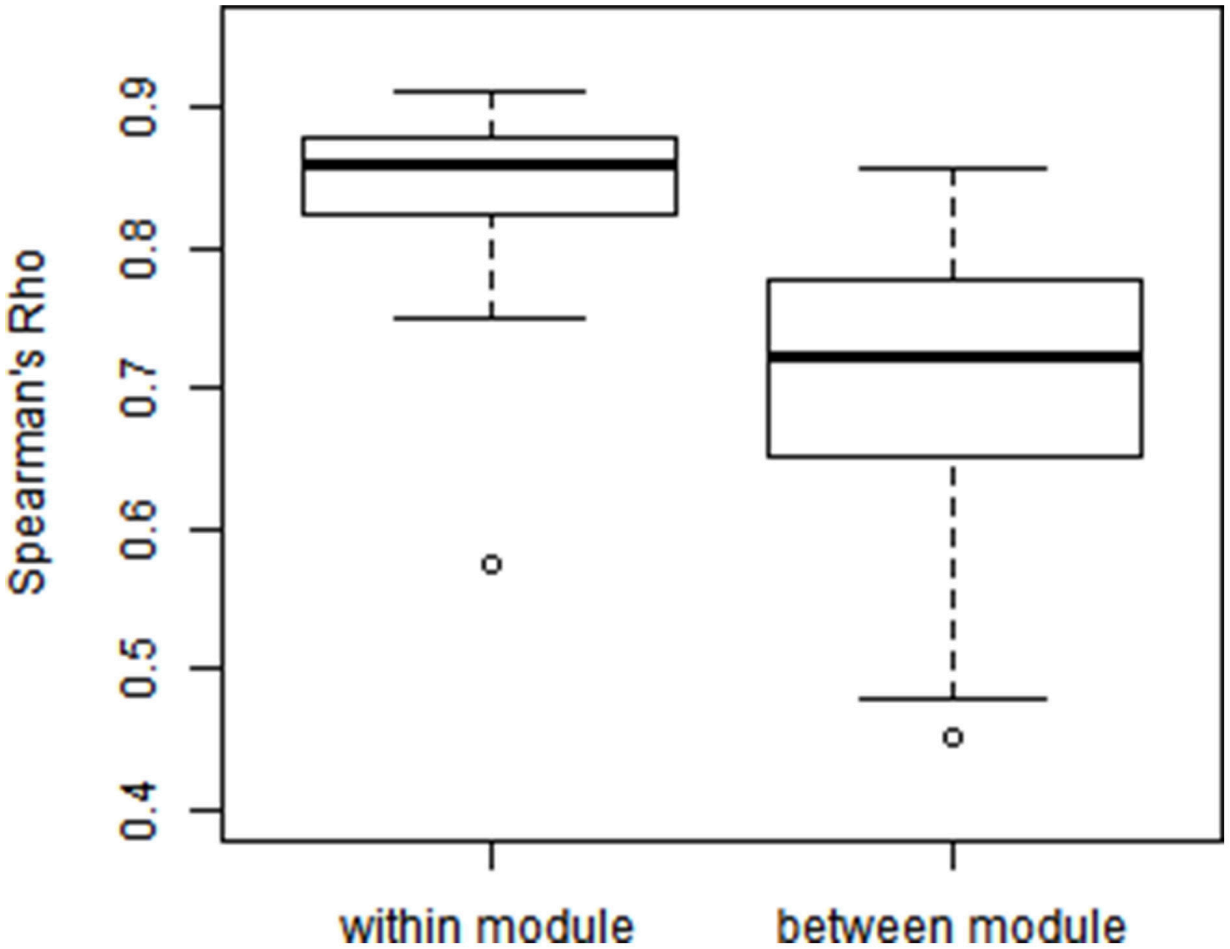
**Modulewise Spearman's rank correlation between partial correlation and full correlation**. We calculated Spearman's rank correlation between partial correlation and full correlation within each of the 55 functional module pairs (Smith et al., 2009), where the Spearman's rank correlation between the full correlation and partial correlation for within-module edges [Mean(SD) = 0.825(0.098)] was significantly higher than those [Mean(SD) = 0.702(0.103)] for between-module edges (*p* = 0.003).

We examined the consistency between partial correlation and full correlation findings across all edges in the network. Since the measures have different scales, we utilized Spearman's rank correlation coefficient (Spearman's Rho) to measure their association at all edges. As shown in Figure 8, the Spearman's Rho between the full correlation and partial correlation for within-module edges (Mean ± SD = 0.825 ± 0.098) was significantly higher than those for between-module edges (Mean ± SD = 0.702 ± 0.103; *p* = 0.003). This demonstrates that partial correlation and full correlation were more consistent for within-module edges compared to between-module edges.

**Figure 9:**
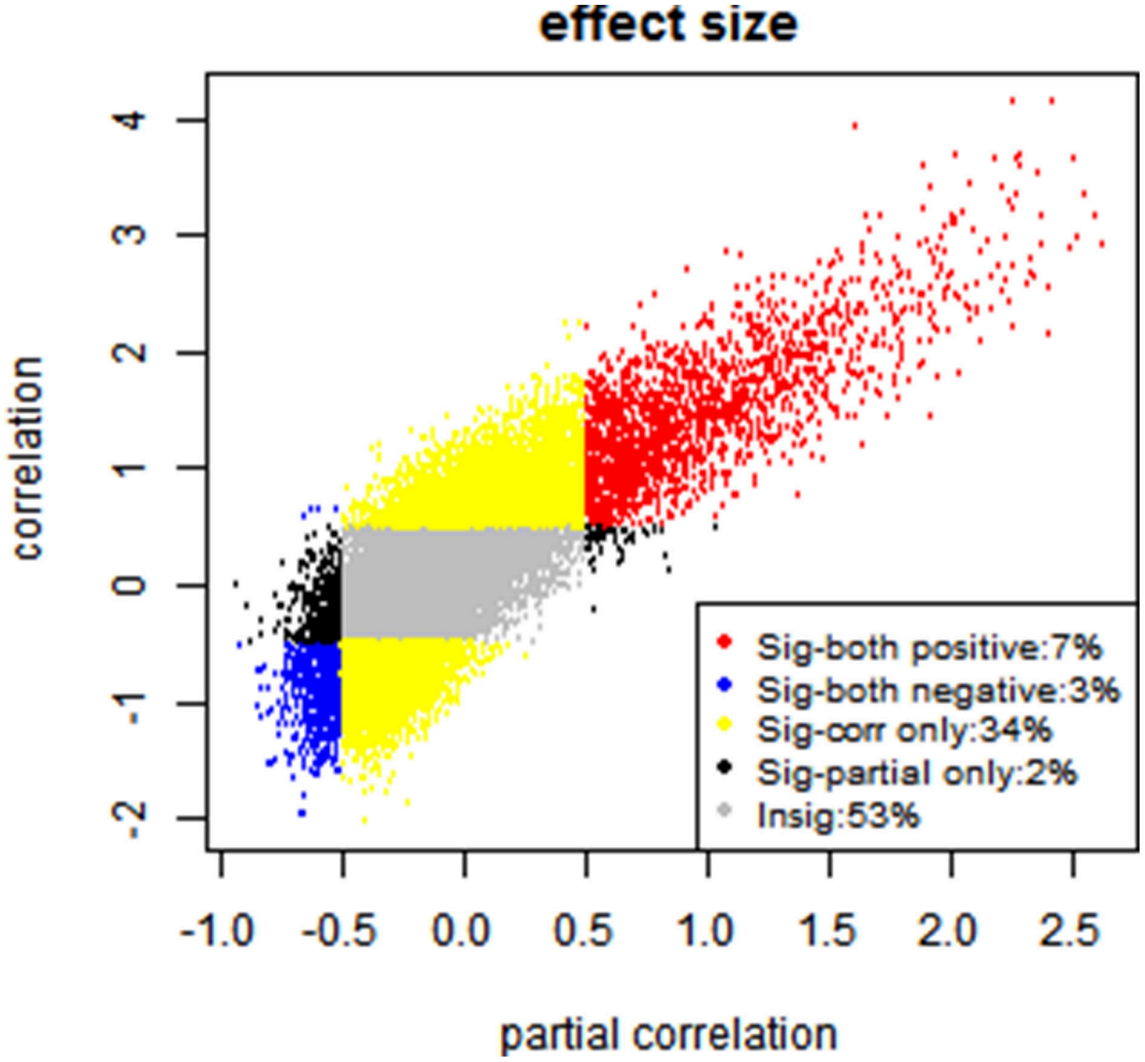
**Effect size-based analysis**. Each edge is classified into four categories based on its effect size under partial correlation and full correlation, where the significant threshold for absolute effect size is set to be 0.5.

Furthermore, since researchers are mostly interested in significant connections, we examine the consistency between the partial correlation and full correlation for these significant edges. Given the large sample size of the PNC data, we have high statistical power to detect even very small deviations from zero in the correlations. Therefore, even edges with very small effect size demonstrated highly significant *p*-values in hypothesis testing. Therefore, we used the effect size instead of *p*-values for thresholding purpose. Specifically, we first performed the Fisher's Z transformation on both the partial correlation and full correlation values. We then calculated the effect size for the connectivity at each edge by diving the mean of z-transformed full correlations or partial correlations to its standard deviation (Kemmer et al., 2015). The effect sizes ranged from −2 to 4 for full correlation and −1 to 2.5 for partial correlation (see Figure 9). We then defined significant edges as those with an effect size of greater than 0.5.

After the thresholding to define the significant edges, each edge is classified into one of the following four categories: (A) significant in partial correlation but insignificant in full correlation (2%); (B) significant in full correlation but insignificant in partial correlation (34%); (C) significant in both (10%); (D) insignificant in both (53%), shown in Figure 9. Moreover, we evaluated the sign consistency between the full correlation and partial correlation on the edge level. The percentage of edges with sign consistency within each of those four categories are A: 83.54%, B: 86.52%, C: 100% and D: 66.05%.

**Figure 10:**
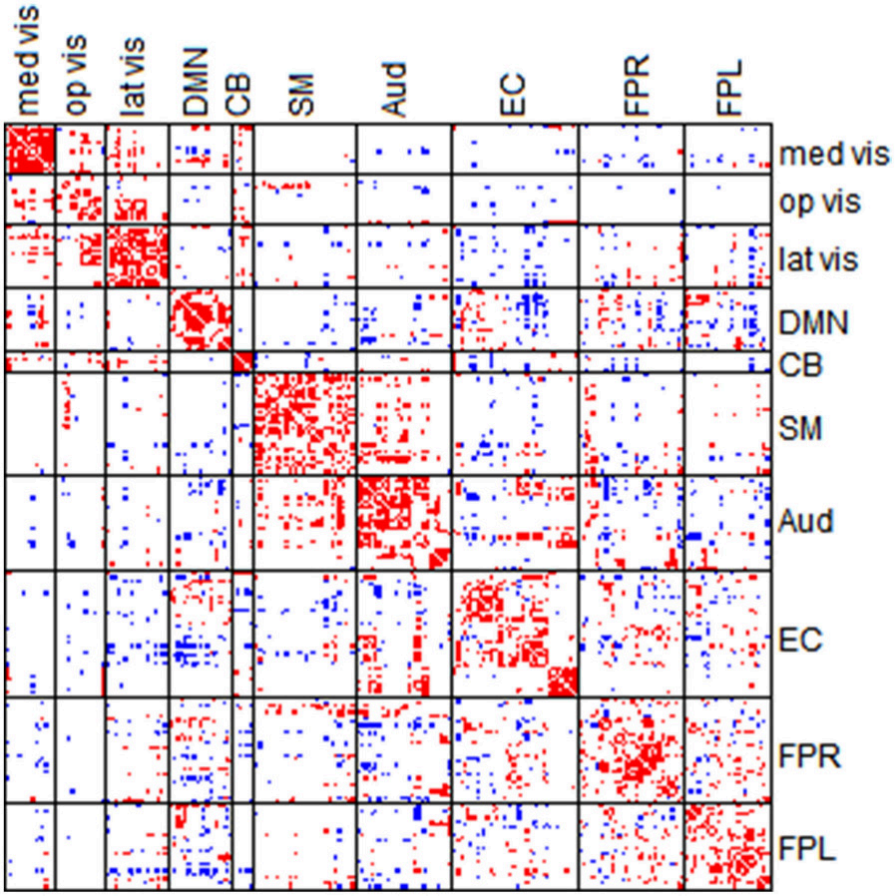
**Edges in category C**. Category C contains the significant edges in both partial correlation and full correlation (absolute effect size > 0.5); Red denotes positive edges and blue denotes negative edges.

**Figure 11:**
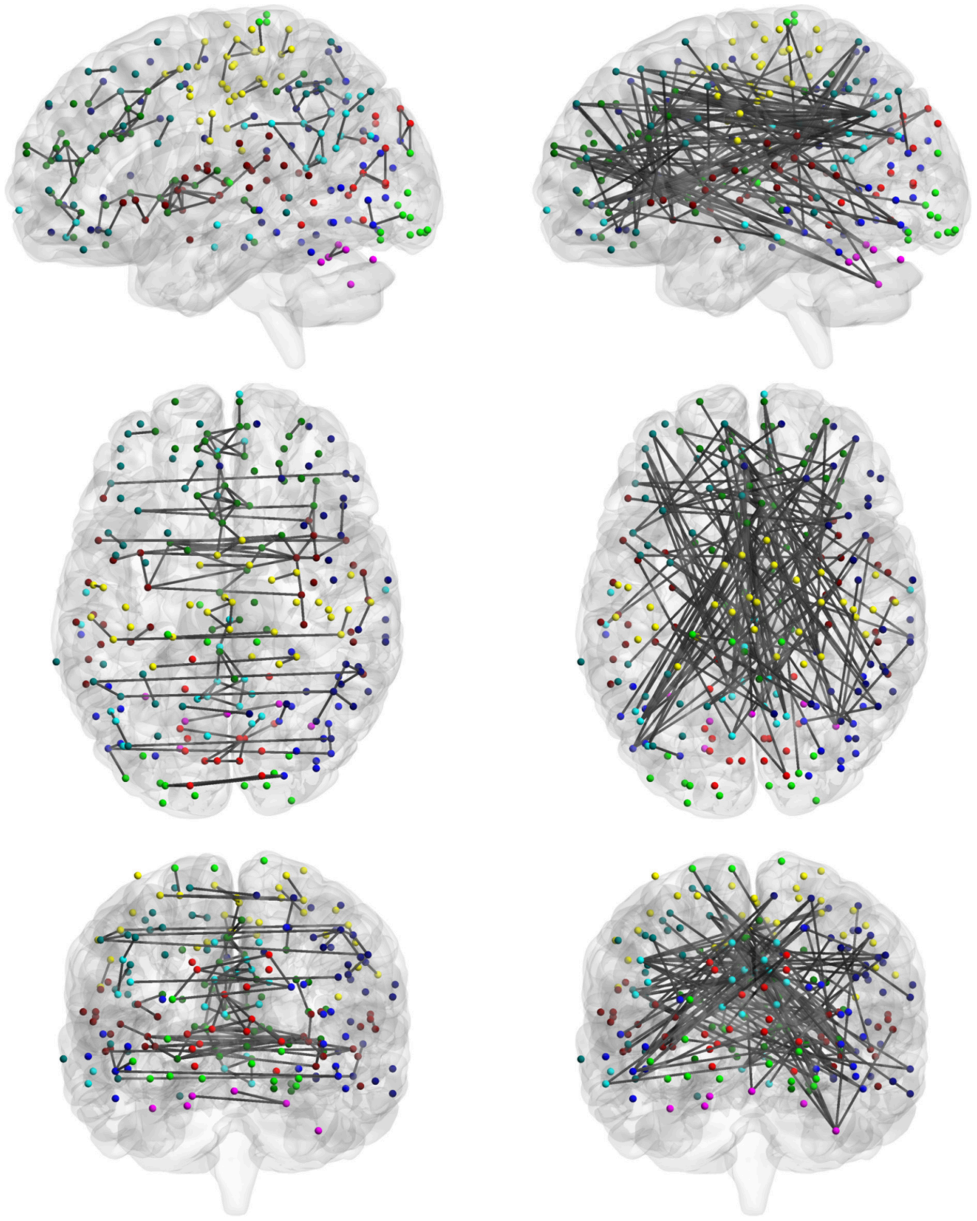
**Highly consistent positive and negative edges between partial correlation and full correlation**. We selected the top 130 positive edges and top 130 negative edges with an absolute effect size larger than 0.5 in both partial correlation and full correlation. (left: positive; right: negative).

Among the four categories, category C reveals the significantly consistent edges based on full correlation and partial correlation. Figure 10 displays edges mapped to the module-wise blocks. Results show that consistently significant positive edges were more concentrated at the within-module regions and consistently significant negative edges were more concentrated at the between-module regions. In particular, we found that considerable consistently negative connections were observed between the default mode network and other modules, especially with the executive control module. To provide better visualization of these consistently significantly edges, we selected the top 130 positive edges and top 130 negative edges from category C and mapped them onto the brain (Figure 11). An important observation from Figure 11 is that the strongest positive connections based on both partial correlation and full correlation were the connections between homologous brain locations in the left and right hemisphere. This finding is consistent with some previous findings based on PET resting-state data collected on rats which also showed that the largest partial correlation coefficients in rate brain were between homologous brain regions (Horwitz et al., 1984). Another important observation from Figure 11 is that the strongest negative connections based on both partial correlation and full correlation tend to have longer spatial distance than strongest positive connections, which is consistent with the previous findings showing that the percentage of negative functional connectivity and spatial distance are significantly correlated (Chen et al., 2011).

We further examined edges in category B, which represented edges that were significant based on full correlation but insignificant based on partial correlation. We examined the proportion of category B edges in each of the module-wise blocks and found that these inconsistent edges were more likely to be observed in between-module connections than for within-module connections. In particular, we found that the following three between-module pairs showed the highest inconsistency between the marginal and direct connectivity: Med vis and FPL for which 56% of all edges between these two networks were in category B, that is only significant based on full correlation; Lat vis and EC for which 50 of all edges between them were in Category B.

### Comparison between network connectivity using partial correlation matrix based on different *Dens* level

In this section, we explore the difference in the estimated direct connectivity based on the proposed partial correlation method using different levels of sparsity control. Specifically, we compared partial correlation estimates obtained with $\lambda_{\text{platu}}^{*}$where minimum sparsity control was applied vs. partial correlation estimates obtained with $\lambda_{0.45}^{*}$ where some sparsity regularization were applied such that the partial correlation matrix reached about 45% of the maximum density level.

**Figure 12:**
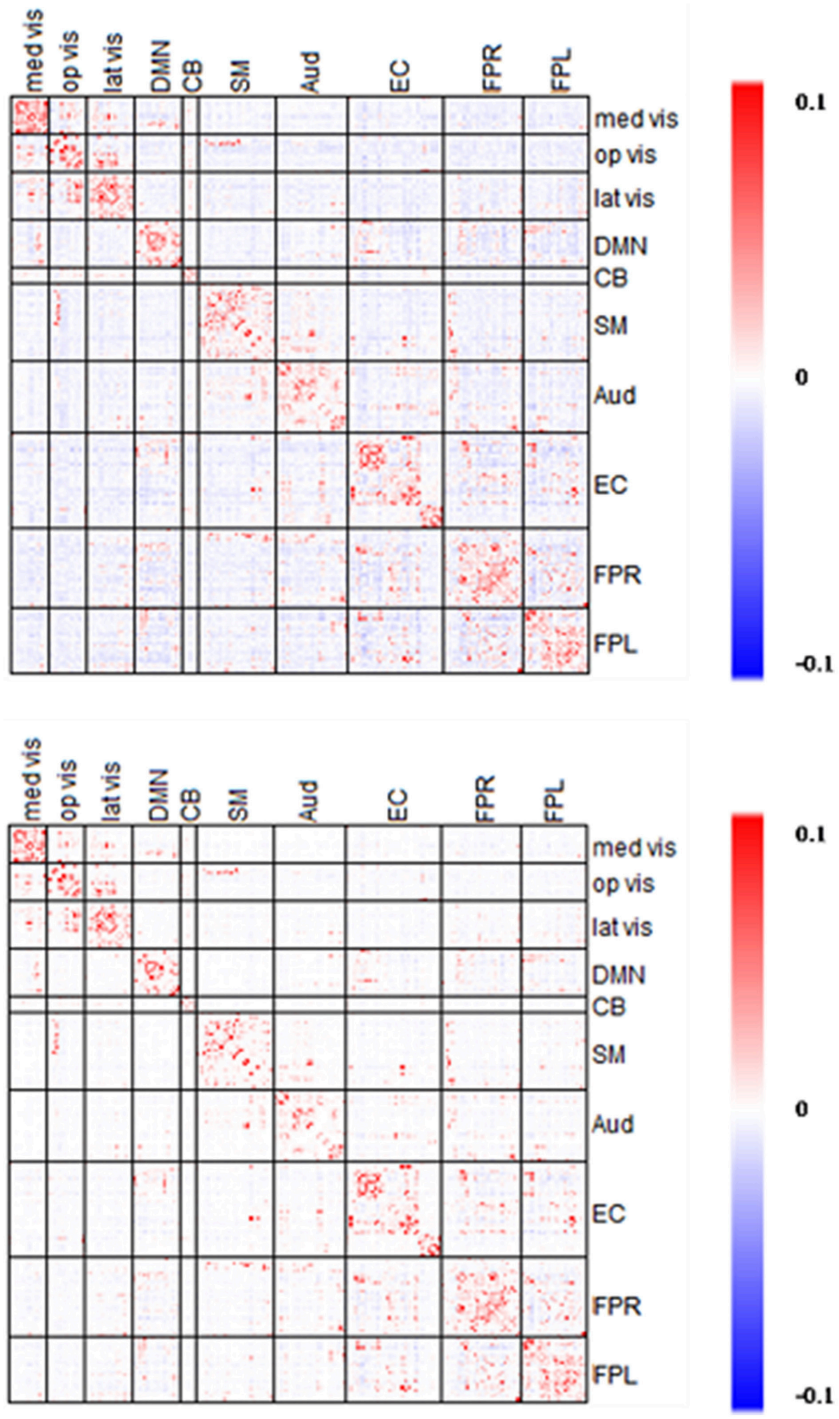
Averaged edgewise λ^*^_platu_- and λ^*^_0.45_-based Partial Correlation matrices. The symmetric 232 × 232 partial correlation matrices based on λ^*^_platu_ **(top)** and λ^*^_0.45_ **(bottom)**. Red indicates the positive edges and blue indicates the negative edges.

The estimated partial correlation matrices based on $\lambda_{\text{platu}}^{*}$ and $\lambda_{0.45}^{*}$ are presented in Figure 12. As expected, the partial correlation matrix based on $\lambda_{0.45}^{*}$ was sparser than that based on $\lambda_{\text{platu}}^{*}$. Furthermore, in the between-module regions the majority of negative (blue) connections under $\lambda_{\text{platu}}^{*}$ disappeared using $\lambda_{0.45}^{*}$, while in the within-module regions the positive (red) connections under $\lambda_{\text{platu}}^{*}$ were retained using $\lambda_{0.45}^{*}$. Marginally, the partial correlations ranged between −0.03 to 0.18 based on $\lambda_{\text{platu}}^{*}$ and −0.02 to 0.22 based on $\lambda_{0.45}^{*}$. Therefore, the limit of the estimated correlations shrank slightly in the negative edges but increased in the positive edges.

**Figure 13:**
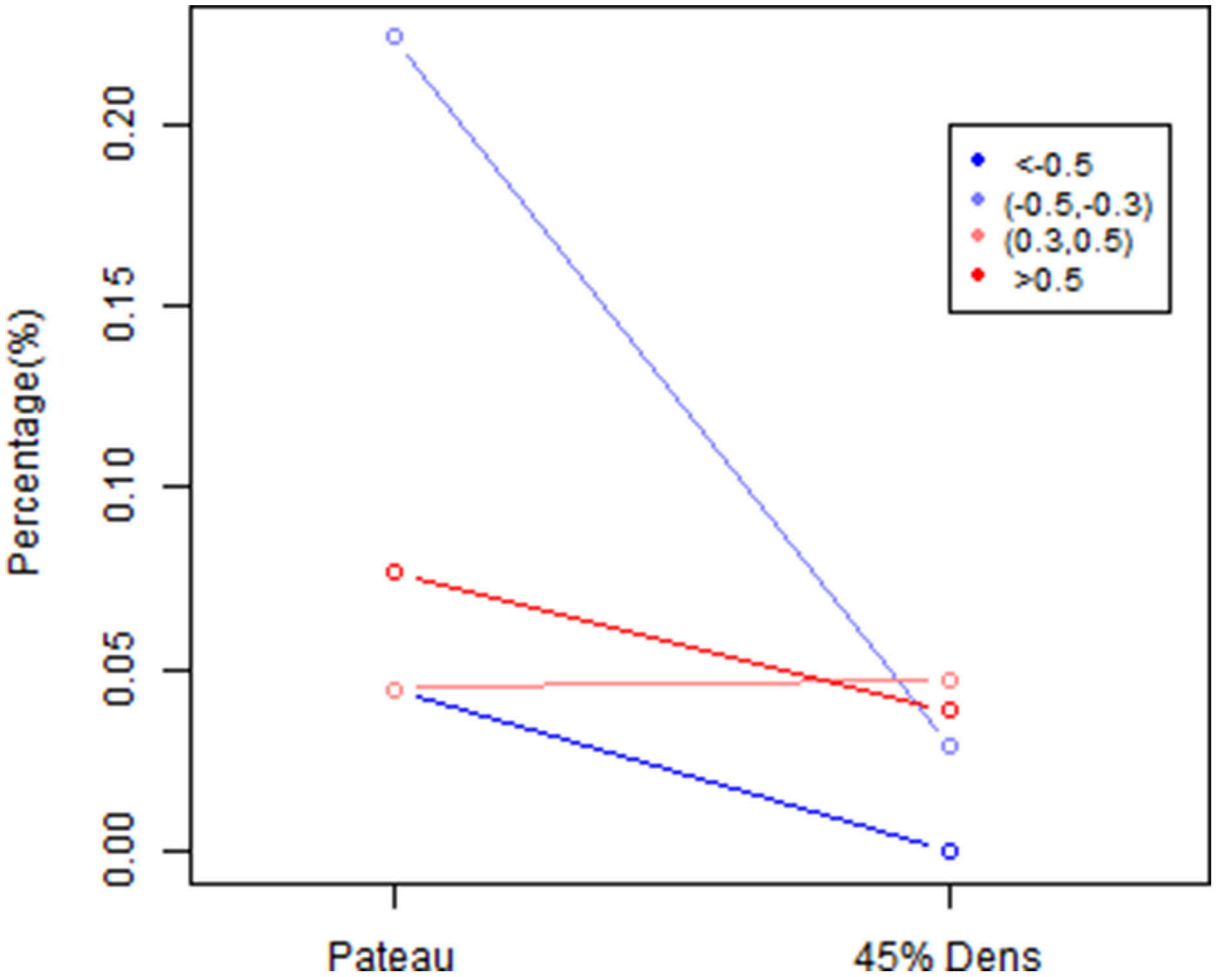
The percentage difference based on size effect between λ^*^_platu_- and λ^*^_0.45_-based partial correlation matrix. Edges are classified into 4 categories based on size effect: significant positive : >0.5, significant negative: < −0.5, moderate positive: (0.3,0.5) and moderate negative (−0.5, −0.3).

To further explore this shrinkage effect, we examined the edges with an absolute effect size larger than 0.3. As shown in Figure 13, the majority of the negative edges with medium (0.3–0.5) to large (>0.5) effect sizes disappeared under a stronger sparsity control, whereas the percentage of the negative edges with medium effect sizes decreased from 22.4 to 2.9%, and the percentage of the negative edges with large effect sizes was decreased to 0.04%. However, the positive edges with medium to large effect sizes were mostly retained under a stronger sparsity control. These results suggest that for positive edges, the edges with medium and large effect size remained fairly robust under shrinkage. However, the negative edges were more likely to disappear under the stronger sparsity control, so the shrinkage effects were much stronger for negative edges than for positive edges. This result suggests that the when applying more sparsity regularization in our proposed procedure, we will still maintain the ability to detect the significant positive edges while the negative edges would experience more shrinkage in the estimates. Again this may be mainly due to the fact that a lot of the negative connections observed in rs-fMRI data were not due to direct connection or neurophysiological effect but rather due to artifacts from imaging processing or biological reasons (Chen et al., 2011; Goelman et al., 2014).

## Discussion

In this paper, we propose a more efficient and reliable statistical method for estimating partial correlation in brain network modeling, which provides a useful tool to investigate direct brain functional connectivity. Compared to existing methods used in the neuroimaging community, the proposed method is shown to be more reliable and computationally efficient. Another major advantage of this technique is that it is scalable to large-scale brain networks with a large number of nodes, for which the existing methods often fail to generate reliable network estimates. Thus, the proposed method can provide a powerful tool for investigating whole brain connectivity in both task-related as well as resting state fMRI studies.

When estimating the partial correlation matrix under the regularization framework, a major challenge is how to select an appropriate tuning parameter to control the sparsity level. Existing selection approaches are often made based on subjective choices or by considering only a few candidates. We propose a new *Dens*-based selection method which considers a series of values across the range of the tuning parameter, and we evaluate the proposed *Dens* criterion for the estimated precision matrix at each value. Hence, we can have a more comprehensive picture of the whole profile of the criterion function across the range of the tuning parameter. Based on the Dens profile, users can now have better understanding on the implications on the sparse level of the estimated networks based on different tuning parameters. Thus, they can make more informed choices of the tuning parameter based on the desired *Dens* level they would like to achieve in the estimated partial correlation matrix. Our proposed *Dens*-based selection method is also {} much faster than the existing selection methods. This will allow users to perform the selection process across many or even all subjects to evaluate the consistency in the selection of the tuning parameter across subjects and to select a common tuning parameter that has good performance across different subjects. In comparison, some of the existing selection methods, such as the cross-validation based method, are very time consuming and hence it is very difficult to conduct such consistency checks across a large number of subjects. Our results from the PNC data showed that the proposed selection procedure leads to a fairly consistent choice of the tuning parameter across different subjects. Therefore, we can apply the same regularization across all subjects, which facilitates performing group analysis of the partial correlations.

When comparing the partial correlation-based and full correlation-based connectivity matrices, we note that the partial correlation removed considerable marginal correlations found in the full correlation matrix that may be due to non-neurophysiological confounding factors. For example, in the partial correlation matrix, many of the significant marginal connections in between-module pairs were not present suggesting these connections between different brain modules were likely caused by global effects or common connection to a third party (Smith et al., 2011; Smith, 2012). Furthermore, the full-correlation-based connectivity matrix demonstrated considerable amount of negative functional connectivity in between-module pairs. Neuroimaging literature has shown that many negative connection findings in rs-fMRI may be caused by non-neurophysiological reasons such as artifacts from global signal removal or inhomogeneous cerebral circulation across the brain (Chen et al., 2011; Goelman et al., 2014). There are considerable controversies in terms of origin and interpretations for these negative connections (Giove et al., 2009; Murphy et al., 2009; Weissenbacher et al., 2009). Hence, many network analyses simply ignore all negative connection (Buckner et al., 2009; Meunier et al., 2009; Satterthwaite et al., 2015). When applying the partial correlation to investigate the direct functional connectivity, we observed that many of the negative connections disappear and those that remain tend to be well-established negative connections such as those between default mode network and other networks. Moreover, based on our *Dens*-based method, we demonstrated that the moderate negative connections were less robust than the positive connections and the strong negative connections, further indicating that a lot of the moderate negative functional connectivity may be caused by non-neurophysiological reasons. By using the proposed partial correlation method with appropriate sparsity control, we can potentially perform meaningful network analysis for negative connections as well in brain network modeling.

An R package “DensParcorr” for implementing the proposed statistical methods can be downloaded from CRAN and the website of Center for Biomedical Imaging Statistics (CBIS) of Emory University.

## Author contributions

YW and YG developed the methodology and performed the analysis of PNC data; YW, YG, and KJ developed and conducted the simulation study; YW and PK preprocessed the data; YW, YG, PK, and JK wrote the manuscript.

### Conflict of interest statement

The authors declare that the research was conducted in the absence of any commercial or financial relationships that could be construed as a potential conflict of interest.
